# Diversity of a bacterial community associated with *Cliona lobata* Hancock and *Gelliodes pumila* (Lendenfeld, 1887) sponges on the South-East coast of India

**DOI:** 10.1038/s41598-020-67717-9

**Published:** 2020-07-14

**Authors:** Ramu Meenatchi, Pownraj Brindangnanam, Saqib Hassan, Kumarasamy Rathna, G. Seghal Kiran, Joseph Selvin

**Affiliations:** 1 Department of Microbiology, School of Life Sciences, Pondicherry University, Puducherry, India; 2 Centre for Bioinformatics, School of Life Sciences, Pondicherry University, Puducherry, India; 3 Department of Food Science and Technology, School of Lifesciences, Pondicherry University, Puducherry, India

**Keywords:** Microbial communities, Environmental microbiology, Ocean sciences

## Abstract

Marine sponges are sources of various bioactive metabolites, including several anticancer drugs, produced mainly by sponge-associated microbes. Palk Bay, on the south-east coast of India, is an understudied, highly disturbed reef environment exposed to various anthropogenic and climatic stresses. In recent years, Palk Bay suffered from pollution due to the dumping of untreated domestic sewage, effluents from coastal aquaculture, tourism, salt pans, cultivation of exotic seaweeds, and geogenic heavy-metal pollution, especially arsenic, mercury, cadmium, and lead. Low microbial-abundant sponge species, such as *Gelliodes pumila* and *Cliona lobata*, were found to be ubiquitously present in this reef environment. Triplicate samples of each of these sponge species were subjected to Illumina MiSeq sequencing using V3–V4 region-specific primers. In both *C. lobata* and *G. pumila,* there was an overwhelming dominance (98 and 99%) of phylum *Candidatus Saccharibacteria* and *Proteobacteria,* respectively*.* The overall number of operational taxonomic units (OTUs) was 68 (40 and 13 OTUs unique to *G. pumila* and *C. lobata*, respectively; 15 shared OTUs). *Alphaproteobacteria* was the most abundant class in both the sponge species. Unclassified species of phylum *Candidatus Saccharibacteria* from *C. lobata* and *Chelotivorans composti* from *G. pumila* were the most abundant bacterial species. The predominance of * Alphaproteobacteria* also revealed the occurrence of various xenobiotic-degrading, surfactant-producing bacterial genera in both the sponge species, indirectly indicating the possible polluted reef status of Palk Bay. Studies on sponge microbiomes at various understudied geographical locations might be helpful in predicting the status of reef environments.

## Introduction

Sponges (phylum: Porifera) are sessile filter feeders and are regarded as holobionts, which comprise 35–40% of microbial communities in their mesohyl^1–3^. Based upon the abundance of these microbial communities, sponges are broadly classified into two groups: high microbial abundance (HMA) sponges and low microbial abundance (LMA) sponges. Apart from bacteria, numerous other prokaryotic and eukaryotic monocellular organisms are reported to be present in the mesohyl of marine sponges^4^. Various bioactive metabolites have been isolated from marine sponges, and some of these bioactive compounds act as anticancer agents and have been approved by the Food and Drug Administration (FDA) for anticancer therapies. Among the four sponge classes reported, *Demospongiae* is considered to be the most abundant of all^5,6^.

Sponges are considered to have been in association with microbes since the Precambrian period, and this relationship has immensely helped the ecological succession of this ancient metazoan. This relationship is considered to be transient because the inflow of seawater into the sponge tissues does not allow any particular community of bacteria to remain stagnant (via filter-feeding activity). However, sponges have also been reported to harbour core microbial communities (specific to sponges) that are in a symbiotic relationship with various sponge species irrespective of the geographical location^7–9^. However, in a recent study by Cleary et al., microbes associated with sponges were no longer considered to be as sponge-specific as assumed earlier^10^.

Studies on sponge microbial communities are important for predicting the health of sponges^11^ in a particular environment as they directly represent the specific type of microbes present during the sampling period (indicative of polluted sites or dumpsites). An increase in the abundance of particular species or classes of a bacterial community can indirectly indicate a problem that reef managers should try to evaluate. Globally, marine habitats are experiencing various natural and anthropogenic stresses in recent years, such as increase in sea surface temperature and the amount of plastic and other non-degradable waste dumped into oceans^12–14^.

The bacterial diversity of two sponge species, *Cliona lobata* and *Gelliodes pumila*, in the reefs of Palk Bay belonging to the class of Demospongiae were explored in this study. Functional prediction through KEGG (Kyoto Encyclopedia of Genes and Genomes), BiosurfDB and KO (KEGG Orthology) analyses found the involvement of hydrocarbon and biosurfactant degrading genes^15^ and also supposed to possess genes involved in carbohydrate, amino acid, protein and nucleic acid metabolism. Due to its close proximity to the seashore, Palk Bay is one of the highly disturbed reef environments. In recent years, Palk Bay has been facing both natural and anthropogenic stresses, mainly due to domestic run-offs from households and industries. Thus, this study attempts to report the bacterial diversity of two commonly available sponge species in the highly disturbed reefs of Palk Bay, on the south-east coast of India, through a comprehensive 16S rRNA metagenomic analysis and highlights the abundance of different bacterial phyla, especially the class *Alphaproteobacteria*, and their contribution to the sustainability of these two sponge species.

## Results

### Taxonomic identification of sponges

**Figure 1:**
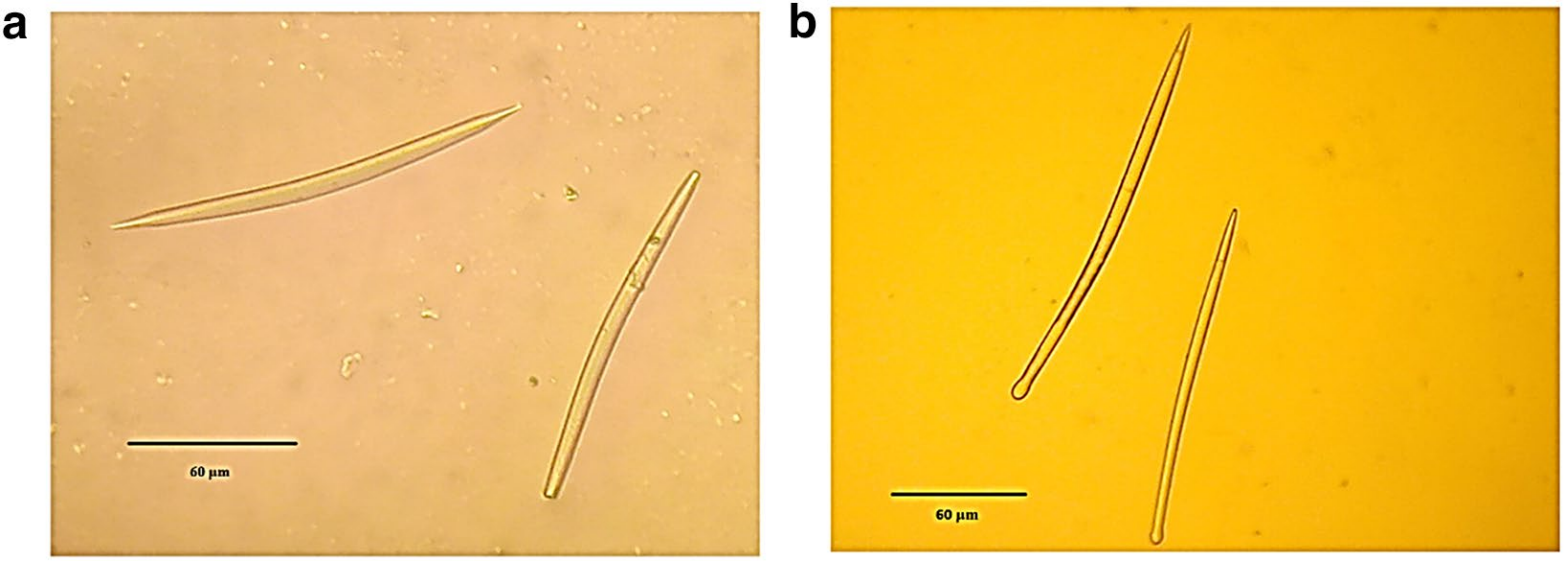
Spicule morphology of sponges *Gelliodes pumila* (**a**) and *Cliona lobata* (**b**)*. G. pumila* shows characteristic oxeas spicule morphology and *C. lobata* shows characteristic tylostyle spicule morphology.

Two sponge species collected from Palk Bay for this study were found to be *Gelloides pumila* (Lendenfeld, 1887) and *Cliona lobata* (Hancock, 1849) based on the morphology and spicule structures. *G. pumila* (class: Demospongiae, order: Haplosclerida, family: Niphatidae, genus: *Gelliodes*, species: *G. pumila* (Lendenfeld, 1887)) was found to be pale grey in colour with large oscules, and the spicules were seen as slightly curved oxeas (size: 110–130 µm). The sponge *C. lobata Hancock* (class: Demospongiae, order: Clionaida, family: Clionaidae, genus: *Cliona*, species: *lobata*) was found to be yellowish brown in colour, and the microscopic examination of the spicules showed characteristic tylostyles with subterminal heads with a size of 120–150 µm (Fig. 1).

### Metadata analysis

A total of about 2.6 million sequence reads with an average length of 602 bp were obtained by Illumina MiSeq sequencing of the sponge metagenome amplicon library (two sponge species with triplicate samples for each) subjected to filtering through QC pipelines. The quality reads of both sponges had a Phred score of 37 and a GC-content range of 51–59%, and the reads were devoid of per base N and Kmer/adapter contents (Table 1).

**Table 1:** Sequence reads and QC results.

	*Gelliodes pumila*			*Cliona lobata*		
Reads	1,260,568			1,390,656		
OTUs	55			28		
Abundance count	631,069			508,914		
Samples	Gr1	Gr2	Gr3	Red1	Red2	Red3
Reads	341,780	369,260	549,528	506,834	427,826	455,996
Total sequences	170,890	184,630	274,764	253,417	213,913	227,998
Phred score	37	37	37	37	37	37
Per base GC content (%)	59	59	51	59	59	59
Per base N content	0	0	0	0	0	0
Sequence length	602	602	602	602	602	602
Adapter content	0	0	0	0	0	0

Using SILVA, single-end reads of 301 nucleotides in length were generated per sample with a list of alignment quality thresholds (consisting of the number of OTUs, sequence reads, taxonomic hierarchy, etc.) for both sponge species, *G. pumila* (represented as Gr1, Gr2, and Gr3) and *C. lobata* (represented as Red1, Red2, and Red3). The results of the SILVAngs analysis performed for the sponge samples were given in Supplementary Data S1. With the help of UPARSE, the accuracy results were improved with far fewer OTUs as compared to the results obtained with the SILVAngs tool. From the metagenomic cumulative sum scaling (CSS) data sets of all six samples representing the two sponge species, a total of 68 non-redundant OTUs were identified from 100 redundant OTUs using the UPARSE tool. The triplicate samples of the two sponge species, *G. pumila* and *C. lobata*, yielded overall total abundance counts of about 6,31,069 and 5,08,914, respectively. Taxonomic classification and OTU clustering by both UPARSE and SILVAngs analysis showed that the most dominant and abundant phylum present in both sponge species was *Proteobacteria* (taxonomic fingerprint: Supplementary Data S1 and S2), and Krona chart (Supplementary Data S3 and S4) analysis in SILVAngs also showed the abundance of *Proteobacteria*.

### Sample distribution and uniformity

In the rarefaction curve, the sample size distributions regarding the number of OTUs between the six samples were non-homogeneous. The number of OTUs in *C. lobata* was found to be less than 30 (with 0–180,000 sample sizes), and each of the triplet samples of *C. lobata* shows a near-plateau curve, displaying homogeneity within its samples (Red1, Red2, and Red3). In contrast, G. pumila was found to have 50 OTUs (with 0–2,000,000 sample sizes), representing heterogeneity within the triplicates (Supplementary Fig. S1). The fraction of OTUs obtained from sequence data was quantified by plotting the rarefaction curves for each of the samples. In the comprehensive bacterial profile, the samples Red1, Red2, Red3, and Gr3 show complete plateaus with few OTUs, indicating considerable level of sequencing. New OTUs would be found if these samples were further sequenced. In comparison, samples Gr1 and Gr2 were found to show incomplete plateaus with high OTUs, indicating that further sequencing is required to obtain a complete bacterial profile.

**Figure 2:**
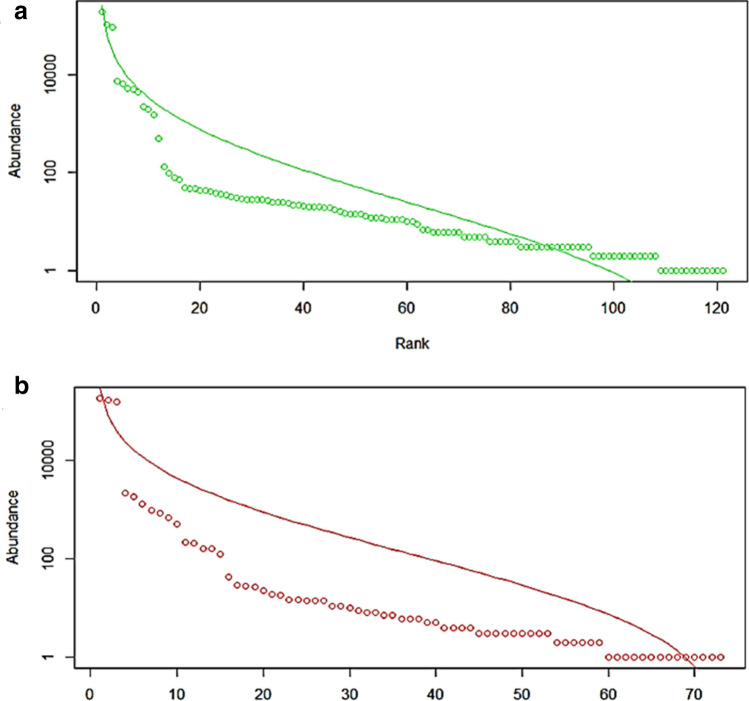
The rank-log abundance graphs of sponge metagenome samples. The conformity to the geometric series distribution of two sponge species namely is showed in (**a**) *Gelliodes pumila* and (**b**) *Cliona lobata.*

In the rank-abundance curve, the conformity of the curves was in accordance with a geometric series distribution of the bacterial species in the two sponge species. In both *C. lobata* and *G. pumila* sponges*,* the overwhelming dominance (98 and 99%) of *Candidatus Saccharibacteria* and *Proteobacteria* over the rest of the phyla was indicative of the displacement of other species by these two phyla (Fig. 2).

### Shared OTUs and abundance

From the comparative study on the overall bacterial community profiles of 68 OTUs, 15 OTUs were found to be shared among the two sponge species, whereas the number of unique bacterial species varied significantly, with 40 species unique to *G. pumila* and only 13 species unique to *C. lobata*.

### Relative abundance: visualisation matrices

**Figure 3:**
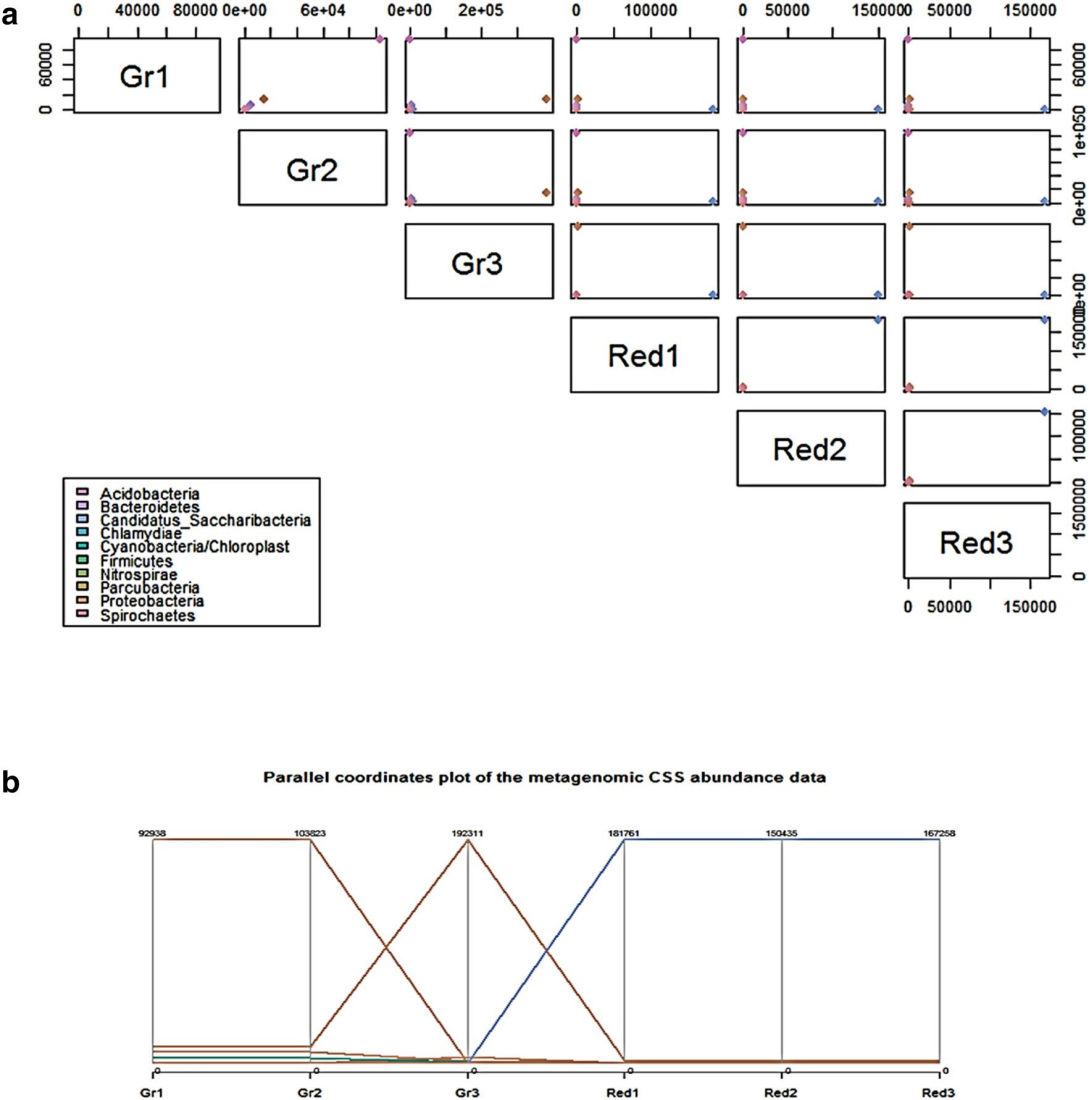
Relative abundance patterns at the level of phyla shown in visualization matrices—scatter plot matrix (**a**) and parallel coordinate plot (**b**). Scatter plot matrix showing the abundance of phyla between and within six samples belonging to two sponge species. The resulting matrix illustrated the multiple distance abundance measures. Based on the abundance values, 68 OTUs of different datasets (samples) dimension has been reduced and the best scatter plot is projected by filters. Parallel coordinate plot points the highest abundance count in each sample of two sponge species and highlight the abundance of phyla *Proteobacteria* (orange), *Candidatus Saccharibacteria* (blue). Relative abundance counts of *G. pumila* samples were 92,938 in Gr1, 103,823 in Gr2 and 192,311 in Gr3 whereas, it was 181,755 in Red1, 150,435 in Red2, and 167,255 in Red3 in *C. lobata*.

The 16S rRNA metagenomic abundance data in a scatter plot matrix (SPLOM), depicting the multidimensional comparison of sponge triplet samples, showed the highest abundance of *Proteobacteria* in *G. pumila* and *Candidatus Saccharibacteria* in *C. lobata* (Fig. 3a). On the basis of the abundance count, the most abundant phylum plotted for *G. pumila* was *Proteobacteria*. However, the most abundant species were *Labrenzia marina* for the Gr1 and Gr2 samples and *Chelativorans composti* for Gr3. The lowest value plotted on the bottom for *C. lobata* was *Candidatus Saccharibacteria*, and the second most abundant one was *Chelativorans composti* in all *C. lobata* data sets (Red1, Red2, and Red3; Fig. 3b).

### Taxonomic diversity: phylum level

Table 2 depicts the absolute count of each of the samples derived using the UPARSE classifier at the phylum level and its respective overall percentage abundance. In all triplet samples of *C. lobata*, 98% of species abundance (*C. lobata*) was found in the *Candidatus Saccharibacteria*, whereas in *G. pumila* it was dominated by Proteobacteria (≥ 99%). Apart from *Proteobacteria* and *Candidatus Saccharibacteria,* other bacterial phyla accounted for only 1% (*G. pumila*) and 2% (*C. lobata*). The bacterial phyla found in the *G. pumila* sponges were found to be more diverse than those in *C. lobata*, as indicated by the presence of several phyla (*Parcubacteria*, *Nitrospirae*, and *Acidobacteria*) only in association with *G. pumila*, while *Spirochaetes* was only present in the *C. lobata* samples.

**Table 2:** Absolute count and overall percentage of bacterial phyla present in *Gelliodes pumila* and *Cliona lobata* samples.

Phylum	Absolute count of *G. pumila* Samples			Overall percentage-*G. pumila* (%)	Absolute count of *C. lobata* Samples			Overall percentage—*C. lobata* samples (%)
	Gr1	Gr2	Gr3		Red1	Red2	Red3	
*Proteobacteria*	104,388	116,610	199,387	98.84	3,529	2,690	3,093	1.82
*Cyanobacteria/Chloroplast*	1,970	2,203	492	1.09	0	3	3	0.001
*Chlamydiae*	39	48	2	0.02	1	1	0	0.0003
*Parcubacteria*	28	20	0	0.01	0	0	0	0
*Firmicutes*	18	29	0	0.01	2	2	1	0.0009
*Nitrospirae*	18	28	0	0.01	0	0	0	0
*Acidobacteria*	6	5	1	0.002	0	0	0	0
*Bacteroidetes*	3	0	0	0.0007	28	19	42	0.01
*Candidatus Saccharibacteria*	0	5	12	0.0039	181,761	150,435	167,258	98.14
*Spirochaetes*	0	0	0	0	4	2	11	0.003

**Figure 4:**
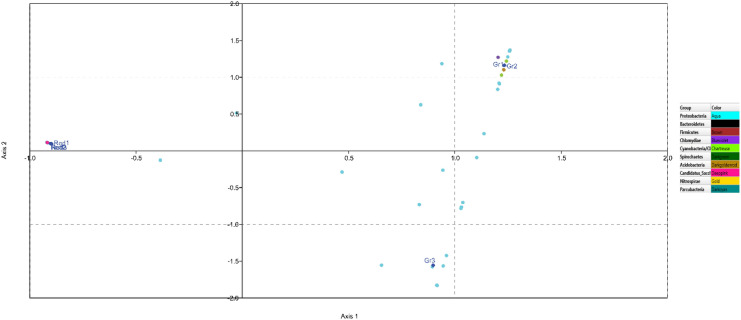
Correspondence analysis showing reproducibility and 16S rRNA profile similarities within *Gelliodes pumila* and *Cliona lobata* samples. CA ordination for triplet microbial community profiles from *G. pumila* (Gr1, 2 and 3) and *C. lobata* (Red1, 2 and 3) represented in dots highlights the species belonging to 10 different phyla in different colour. Triplicate *G. pumila* samples representing Gr1 and 2 found to cluster closely whereas, Gr3 cluster more distantly along the first ordination axis. *C. lobata*-derived triplicates (Red 1, 2 and 3) also show similar diversity pattern. CA plot highlights the abundance of *Proteobacteria* (shown in figure as aqua dots) and the convergence of uncultured *Candidatus Saccharibacteria* phyla represented in deep pink dots. Different colour dots adjacent to the *G. pumila* sponge triplets (Gr1, Gr2 and Gr3) has symbolized its high bacterial diversity as compared to *C. lobata* at phyla level.

The visualisation matrices, such as the abundance-based scatterplot matrix for data sets and the parallel coordinate plot (as previously mentioned), confirmed the abundance of *Proteobacteria* and *Candidatus Saccharibacteria* in both sponge species. Correspondence analysis (CA) is yet another ordination method, somewhat similar to Principle Component Analysis (PCA). It also showed the dominance of *Proteobacteria* in all sponge samples and highlighted the convergence of *Candidatus Saccharibacteria* (Fig. 4).

### Alpha diversity analysis

Triplicate samples of 1,260,568 reads in *G. pumila* were found to contain 55 OTUs, and triplicate samples of *C. lobata* (1,390,656 reads) contained only 27 OTUs. Compared to C. lobata, bacterial diversity of G. pumila seemed to increase proportionally with respect to Shannon index and was found to decrease in case of Simpson index (Table 3). From the overall values of alpha diversity indices and estimators, species richness and evenness was found to be higher in *G. pumila* than in *C. lobata*.

**Table 3:** Characteristics of sponge microbiome alpha diversity and richness.

Alpha diversity indices	Gr1	Gr2	Gr3	Red1	Red2	Red3
Taxa_S	43	49	29	26	24	23
Individuals	106,470	118,948	199,894	185,325	153,152	170,408
Dominance_D	0.7678	0.7677	0.9263	0.9621	0.9649	0.9635
Simpson_1-D	0.2322	0.2323	0.07368	0.03793	0.03507	0.03649
Shannon_H	0.544	0.5439	0.1993	0.1177	0.1144	0.1158
Evenness_e^H/S	0.04007	0.03516	0.04209	0.04326	0.04672	0.04882
Brillouin	0.5432	0.543	0.199	0.1174	0.1141	0.1155
Menhinick	0.1318	0.1421	0.06486	0.0604	0.06133	0.05572
Margalef	3.628	4.107	2.294	2.061	1.926	1.826
Equitability_J	0.1446	0.1398	0.05919	0.03612	0.03601	0.03694
Fisher_alpha	4.245	4.848	2.576	2.302	2.148	2.028
Berger–Parker	0.8729	0.8728	0.9621	0.9808	0.9823	0.9815
Chao-1	43.14	52.33	31.5	29.75	26	26

### SIMPER analysis

SIMPER (similarity percentage) analysis based on the Bray–Curtis distance showed an average dissimilarity for each sample ranging from 7 to 22%. The representative 10 phyla (Table 4) accounted for 81.73% of the overall average dissimilarities.

**Table 4:** SIMPER analysis (Bray–Curtis model).

SIMPER analysis	Gr1	Gr2	Gr3	Red3	Red1	Red2
Av. dissim	20.51	22.89	10.84	10.77	8.91	7.807
Contrib. %	25.09	28.01	13.27	13.18	10.9	9.552
Cumulative %	53.1	28.01	66.37	79.55	90.45	100
Mean *Proteobacteria*	1.93E+03	2.16 E+03	3.69 E+03	57.3	65.4	49.8
Mean *Bacteroidetes*	1.5	0	0	21	14	9.5
Mean *Firmicutes*	4.5	7.25	0	0.25	0.5	0.5
Mean *Chlamydiae*	39	48	2	0	1	1
Mean *Cyanobacteria/Chloroplast*	985	1.10 E+03	246	1.5	0	1.5
Mean *Spirochaetes*	0	0	0	11	4	2
Mean *Acidobacteria*	6	5	1	0	0	0
Mean *Candidatus_Saccharibacteria*	0	5	12	1.67 E+05	1.82 E+05	1.50 E+05
Mean *Nitrospirae*	18	28	0	0	0	0
Mean *Parcubacteria*	28	20	0	0	0	0

Mean percentage abundance values of *Gelliodes pumila* sample (Gr 1, 2 and 3; 22, 20 and 10% dissimilarity, respectively) and *Cliona lobata* samples (Red 1, 2 and 3 shows 10, 8 and 7% dissimilarity, respectively) contributing to differences in each of the samples.

### Taxonomic diversity: species level

**Figure 5:**
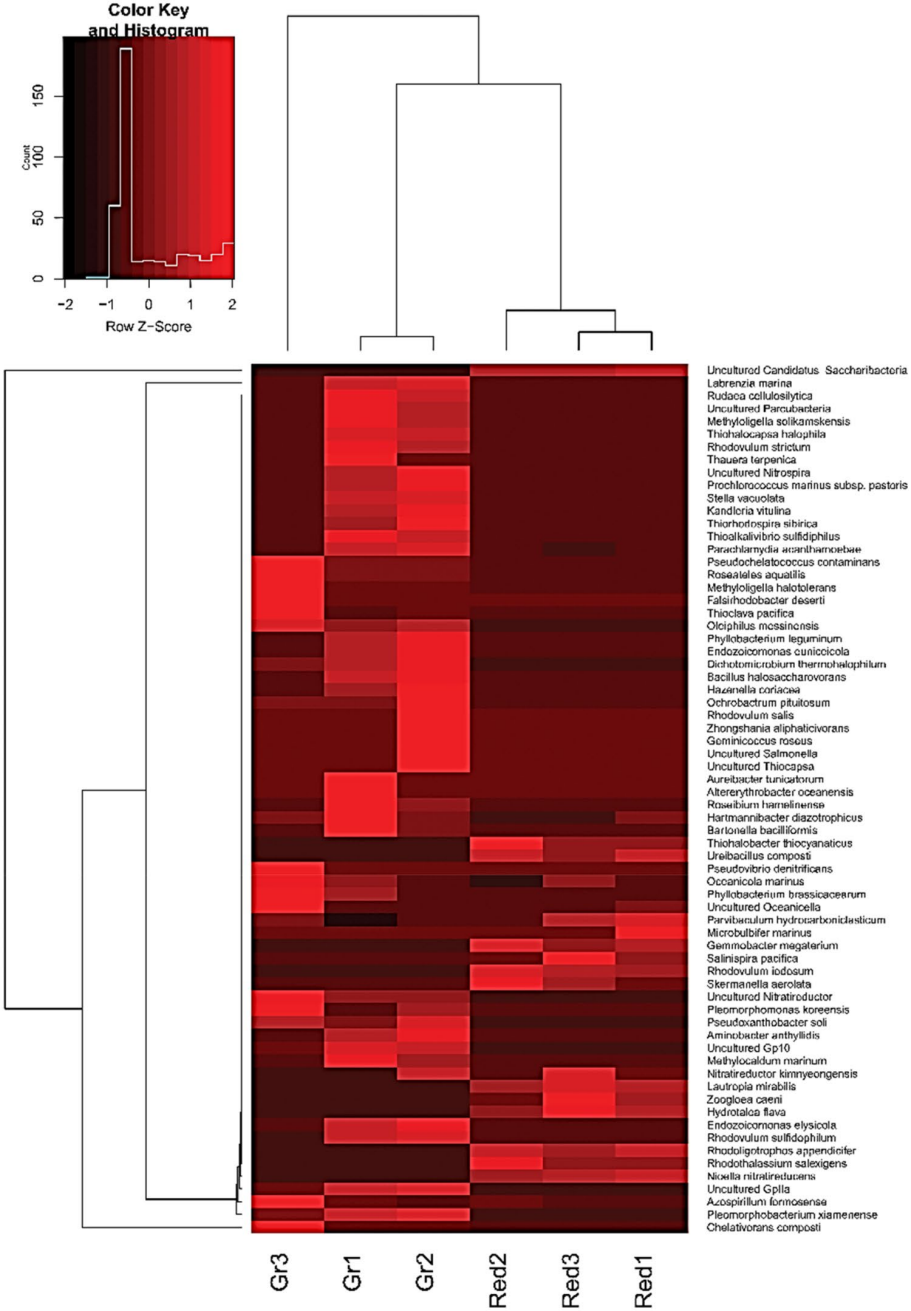
Complete linkage clustering-based heatmap for triplet sample abundance in *Gelliodes pumila* and *Cliona lobata* sponges. Heat map of dendrogram (*y*-axis) inferred from complete linkage clustering-based species abundance values of all samples containing 68 non-redundant species. The *x*-axis shows triplicate samples of each of the sponge species.

Hierarchical clustering (Ward dissimilarity-based matrix) of 68 species of *G. pumila* and *C. lobata* was conducted to predict abundance using the complete linkage clustering method. Uncultured *Candidatus Saccharibacteria* from *C. lobata* and *Chelotivorans composti* from *G. pumila* were found to be the most abundant species (Fig. 5).

### Network analysis-based metagenome hierarchy

**Figure 6:**
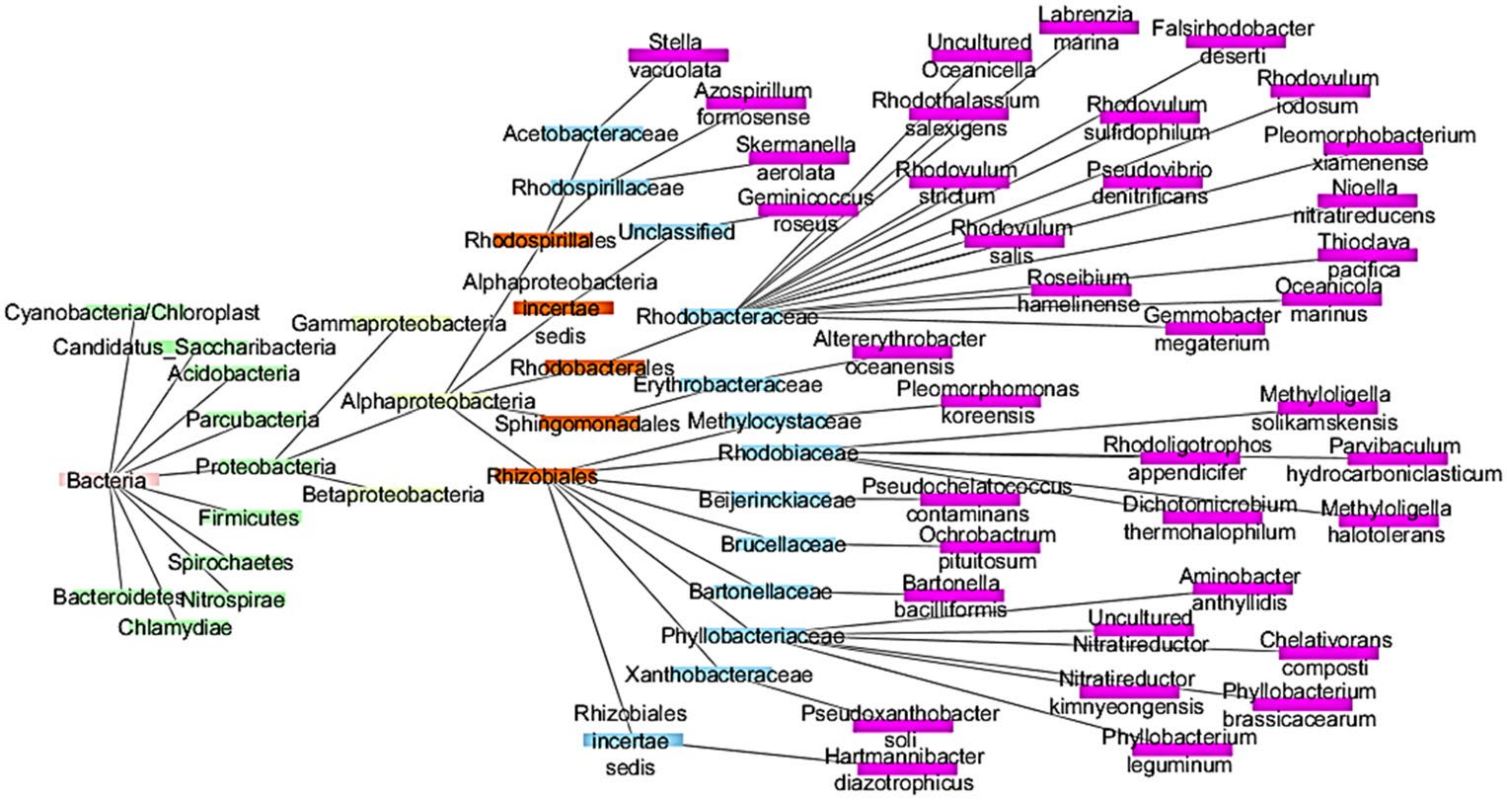
Network based metagenome hierarchy highlights the abundance of *Alphaproteobacteria* (coloured in pink).

Altogether, both sponge species were found to contain 68 bacterial species, excluding the unclassified OTUs. Of these 68 species, 37 bacterial species were found to be belonging to the class *Alphaproteobacteria*, emphasising its dominance over other classes (Fig. 6).

### BioSurfDB and KEGG pathway: functional prediction

Through a BioSurfDB database search, 16% of species (*Labrenzia marina, Methyloligella halotolerans, Methyloligella solikamskensis, Roseibium hamelinense, Skermanella aerolata*, and *Pseudovibrio denitrificans*) belonging to the class *Alphaproteobacteria* were likely to contain functional genes for surfactant production and biodegradation properties (Supplementary Table S1). At the genus level, 51% of the genera (*Altererythrobacter, Azospirillum, Ochrobactrum, Parvibaculum, Rhodovulum*, and *Bartonella*) among the class *Alphaproteobacteria* were anticipated to contain functional genes responsible for xenobiotic (aromatic hydrocarbon and persistent organic pollutant) degradation through KEGG orthology analysis, and they were also supposed to be involved in carbohydrate, amino acid, protein, and nucleic acid metabolism.

## Discussion

Sponges act as filters of reef environments by capturing various planktonic organisms, including microbes^6,16^. Although numerous sponge metagenome studies are underway as a part of the Earth Microbiome project^17,18^, studies using metagenomic analysis of sponges from various understudied reef environments worldwide are of prime importance for predicting the global status of reefs. Palk Bay is a nearshore reef environment highly exposed to various anthropogenic and climatic pressures, such as pollution due to improper waste disposal, coastal run-offs, and trap fishing. Studies on Palk Bay, report shifts in ecological balances, as depicted by algal bloom and *Terpios* attacks^19,20^. As reported by Bell et al.^11,21^ and Easson et al.^22^, alterations in nutrient cycling or ecosystem imbalance can result in a decline of sponge diversity. Accordingly, in Palk Bay, especially in the study location (see “Methods”), the diversity of sponges was negligible, and only two species of sponges were predominant, *G. pumila* and *C. lobate*, both belonging to the class *Desmospongiae.*

Although sponge diversity tends to decrease during imbalances in reef environments due to pollution, some sponges tend to thrive in these habitats by exploiting the properties of various microbial symbionts. Apart from these two sponge species, one of the encrusting sponges identified as *Terpios* was extremely predominant in Palk Bay. It kills most corals by growing over them using an actively growing symbiotic cyanobacterial mat^19,22^. Likewise, free-living sponges also contain bacterial symbionts in their mesohyl, and these symbiotic bacteria shape these sponges so that they can endure these disturbed environments. The bacterial phyla commonly reported to be associated with sponge mesohyl through metagenomic analysis include *Acidobacteria*, *Actinobacteria*, *Chloroflexi*, *Nitrospira*, *Cyanobacteria*, *Bacteriodetes*, *Gemmatimonadetes*, *Planctomycetes*, *Proteobacteria* (Alpha- and Gammaproteobacteria), and *Spirochaetes*^23–26^. In this study, *Candidatus Saccharibacteria* and *Proteobacteria* were found to be dominant over other bacterial phyla. It appears that *Candidatus Saccharibacteria* and *Proteobacteria* are negatively affecting the relative abundance of the other common phyla specific to high microbial abundance sponges, including *Poribacteria*^27^ and *Chloroflexi*^25^. The two sponges included in this study might be belong to LMA sponges because of the absence of *Poribacteria* and *Chloroflexi* phyla (which are unique to HMA sponges, as reviewed by Pita et al. 2018).

The dominance of the Proteobacteria phylum observed in this study has already been widely reported^28^. The potential bacterial symbionts were identified from the shared bacterial community profiles. In this study, there are 15 OTUs common to all samples within the studied sponge population, regardless of environmental conditions. They may be symbiotically maintained by both sponge genera because of metabolic benefits provided by these bacteria^2,29^. The polluted environment was confirmed by the presence of wastewater-treating phyla in both sponges, such as the anoxic phylum Candidatus Parcubacteria^30^, the nitrate-oxidising phylum *Nitrospirae*^31^, the enhanced biological phosphorus-removing phylum *Acidobacteria*^33^, and the phyla that include lipid-degrading microbes, such as Proteobacteria, Bacteroides, Spirochaetes^34^, Firmicutes^35^, Chlamydia^36^, Candidatus Saccharibacteria^37^, and Cyanobacteria^38^. The properties of these sponge-associated bacterial communities may be the reason for the sustainable growth of *G. pumila* and *C. lobata* in Palk Bay, which is a highly disturbed nearshore reef environment prone to various anthropogenic stresses^20^.

The cytoscape network of metagenome hierarchy visualises the dominance of *Alphaproteobacteria*, and through a BioSurfDB database search (https://www.biosurfdb.org), 16% of species belonging to the class *Alphaproteobacteria* were expected to have surfactant production and biodegradation properties at the species level. Through KEGG analysis, 17% of genera belonging to *Alphaproteobacteria* were predicted to have degradation properties (https://www.genome.jp/kegg-bin/show_pathway), which includes highly toxic environmentally persistent organic pollutants, such as dioxin, aminobenzoate, caprolactam, chloroalkane and chloroalkene, chlorocyclohexane and chlorobenzene, ethylbenzene, fatty acid, fluorobenzoate, naphthalene, nitrotoluene, polycyclic aromatic hydrocarbon, and toluene and xylene. KO studies also showed that these bacterial species belonging to the *Alphaproteobacteria* class are involved in carbohydrate, amino acid, protein, and nucleic acid metabolism (https://www.ebi.ac.uk/QuickGO/term). From this study, we concluded the presence of certain bacterial phyla and classes as pollution indicators which can help to gain more insight into the status of reef ecosystems in various understudied geographical locations, such as Palk Bay (on the South-East of India). Further, Palk Bay has also been reported to be a site contaminated with heavy metals^39,40^. In our study, sponge metagenome analysis along with function prediction analysis indirectly indicates the delicate status of Palk Bay reefs by revealing the presence of various pollution-representative bacterial communities within the sponge holobiont. Although the microbiome analysis of seawater samples found near these reef communities was not included in this study for comparison, this sponge microbiome study indirectly indicates the putative vulnerability of Palk Bay reef communities, which might alarm the reef managers to focus on various reef management strategies to conserve this precious reef ecosystem.

## Methods

### Study site and reef status

Palk Bay is a nearshore fringing reef bay situated at the south east coast of India (adjacent to protected reef environment, Gulf of Mannar) between longitudes 79° 17′ 40ʺ E and 79° 8′ E and at latitude of 9° 17′ N with an average depth between 1 and 5 m. Palk Bay is a highly disturbed and unprotected reef ecosystem serving as homeland for several species of marine algae, fishes, turtles, corals, sponges, seagrass, mangroves and so on^41^. Palk Bay suffers because of pollution in recent years due to dumping of untreated domestic sewage, effluents from coastal aquaculture, tourism, salt pans, cultivation of exotic seaweeds, geogenic heavy metal pollution especially with arsenic, mercury, cadmium and lead^39,40,42,43^.

### Sample collection and sponge identification

*Desmospongiae* is the most predominantly found sponge class in Palk Bay, and two most commonly available sponge species belonging to this class were selected for this microbiome study. Around 0.5 g of representative sponge samples (belonging to two commonly available sponge species) were collected by snorkelling (three specimens from each representative sponge species) during May 2016. Sponge samples were transported to the laboratory as two sets, one in DMSO-EDTA saline saturated (DESS)^44^ solution and other in ice (4 °C). For DNA extraction, samples were preserved in DESS solution while transporting to maintain DNA integrity. For sponge identification, the samples were rinsed first with filter-sterilized seawater to remove debris and then stored in ice during transportation. After reaching the laboratory, both the samples were stored at -80 °C until further processing, sponge samples transported in DESS solution were first rinsed with filter-sterilized seawater to remove excess salts and to prevent interference of salts during DNA extraction. All sponge samples were collected from a depth of about 2 m.

Sponge samples (about 0.5 cm) transported in ice were treated with 1.5 ml sodium hypochlorite (30%, v/v) solution for tissue digestion. After 1 h incubation, the tubes were centrifuged at 4,000 rpm for 5 min (Eppendorf) and then washed twice with sterile distilled water to remove cell debris. The suspension was visualized under light microscope to observe the sponge species-specific distinctive spicule morphology. The spicule morphology was recorded and photographed, then the spicule size was measured using KLONK Image Measurement software (version: 19.4.15.0). The two morphologically different sponge samples collected from Palk Bay coded as ‘Gr’ (pale grey in colour) and ‘Red’ (yellowish brown in colour) were identified based on spicule structure reports of Sivaleela^45^ and marine species database (https://www.marinespecies.org).

### Sample processing and metagenomic DNA extraction

Sponge metagenomic DNA was isolated using the DNeasy plant DNA isolation kit (Qiagen GmbH, Hilden) following the manufacturer’s protocol. Totally, six samples of metagenomic DNA were subjected to Illumina Sequencing, in which three samples belong to *G. pumila* (Lendenfeld, 1887)^46^ (coded as Gr1, Gr2, and Gr3) and three samples belong to *C. lobata* Hancock^47^ (coded as Red1, Red2, Red3). The purity of the DNA samples was determined in a NanoDrop ND-2000 spectrophotometer (Thermo Fisher Scientific, USA) and the DNA concentration was quantified by using a Qubit fluorimeter.

### PCR and Illumina next generation sequencing (NGS)

Bacterial V3-V4 hypervariable regions of the 16S rRNA genes were amplified using the specific universal primers 341F, 5′-CCTACGGGAGGCAGCAG-3′ and 518R, 5′-ATTACCGCGGCTGCTGG-3′. PCR amplification was performed using an initial denaturing step of 98°C for 3 min, followed by 20 cycles at 98°C for 30 s, 60°C for 30 s, and 72°C for 30 s, and then an elongation step of 72°C for 5 min. Subsequently, indexing PCR, Illumina sequencing adapters, and dual indexing barcodes were added with limited PCR cycles. The prepared libraries were quantified using qPCR according to the Illumina qPCR quantification protocol guide. To generate a standard curve of fluorescence readings and to calculate the library sample concentration, Roche's Rapid library standard quantification process has been performed. The purified PCR products (library) were pooled into equimolar ratios and paired-end reads were generated on an Illumina GAIIx sequencer. Image analysis and base calling were performed using Illumina Analysis pipeline (Version 2.2). The NGS sequencing services were provided by Bionivid Technology Private Limited, Bangalore, India.

### Metagenomic data manipulation and QC

Raw reads were subjected to pre-processing through quality checking, operational taxonomic units (OTU) clustering, and OTU annotation using scientifically accepted tools and pipelines. QC and filtering of high-quality sequencing data was performed to find the low quality reads, unique reads, de-replication, de-noising removal of singleton and chimera filtering using a Ribosomal Database Project (RDP) Gold UCHIME reference database. To validate the quality of the reads, Galaxy tool version 2.0.1.1 and 0.72 (https://usegalaxy.org/) was used which includes FASTQ joiner (to join paired end FASTQ reads from two separate files into a single read) and FastQC (to give read quality report). The base quality parameters included in this study were Phred score of more than 30, GC content of more than 50% and lack of per base N content and Kmer/adapter content.

### OTU picking, clustering and classification

To generate OTU clusters and hierarchical classification from domain to species, UPARSE^48^ and SILVAngs (Version 1.3.9, https://www.arb-silva.de/ngs/) were used. Abundance for each of the samples were subjected to CSS and normalization of CSS data with abundance cut-off > 5. Krona graphs were plotted using SILVAgns by means of SILVA rRNA database as the source. All the quality checking parameters, OTU picking, classification and clustering were crosschecked with SILVAngs platform as well.

### Statistical analysis

The R packages (BiodiversityR^49,50^, ggplot2^51^, dendextend and vegan) along with machine learning approaches were used for quantification, visualization and analysis of metadata of sponges. This includes diversity indices for diversity measures, relative abundance curve, scatter plot, parallel coordinate plot and rarefaction curve for community measures, and several statistical visualization tests such as linkage clustering, correspondence analysis (CA), heat-maps with cluster dendrogram, cytoscape metagenome network, Simper analysis and Neighbor Joining (NJ) Bray–Curtis tree. In a parallel coordinate plot, the relative abundance of microbial phyla can be visualized in multiple division altogether and every parallel line on this plot represents to the new set of data such as Gr1, Gr2, Gr3, Red1, Red2 and Red3 (samples). Each trajectory across the plot gives the row of 6 metric values obtained by an image; altogether there are 68 such trajectories, so an entire table of 68 × 6 metric values was plotted here. Higher scores were preferred, but the results of the metric/score computations in each column have been independently scaled, so the columns represent the microbial phyla at multiple division. To compare the OTU diversity between *Cliona lobata* and *Gelliodes pumila* samples, the following analysis were conducted using the PAST3 tool^52^. The distances of samples in datasets were calculated in terms of OTU profile, eigen values and eigen vectors of a metrics was applied based on Chi-squared distances, which measures structure dissimilarities between communities; the obtained Chi-squared distances matrix was used to calculate and visualize sample similarities using correspondence analysis. To support the visual analysis of triplet samples of two sponges, two visualization matrices were presented. The abundance-based scatterplot matrix for datasets (six samples) with 68 species that supports the analysis of pairwise relationship between the samples, and also allows the correlation in all possible dimensions. The highest and the lowest levels of taxonomic abundance were represented by parallel coordinate plot.

### Function prediction

The BioSurfDB system database (https://www.biosurfdb.org) was used to model the surfactant production and biodegradation domains using the OTUs classified from the 16S rRNA sequence analysis^53^. For prediction and annotation of functional profile using 16S rRNA gene-based OTU clustering, KEGG database (https://www.genome.jp/kegg-bin/show_pathway) was used to estimate all the functional genes found in the microbiota. The functional genes were annotated by KEGG Orthology (KO: https://www.ebi.ac.uk/QuickGO/term) and KEGG pathway^54^ analyses.

### Nucleotide sequence submission

All 16S rRNA datasets generated through this study were deposited as Sequence Read Archive in NCBI database with Bioproject ID: PRJNA501863 and PRJNA501863.

## Supplementary information

Supplementary file1 (PDF 199 kb)

Supplementary file2 (PDF 228 kb)

Supplementary file3 (PDF 135 kb)

Supplementary file4 (PDF 126 kb)

Supplementary file5 (DOCX 317 kb)
