# Supplementary material for: Diversity of a bacterial community associated with Cliona lobata Hancock and Gelliodes pumila (Lendenfeld, 1887) sponges on the South-East coast of India
Source: Sci Rep. 2020 Jul 14;10:11558. doi: 10.1038/s41598-020-67717-9 (PMC7360593; doi:10.1038/s41598-020-67717-9)
Supplement: Supplementary file 1 — Supplementary file1 (PDF 199 kb) [file 41598_2020_67717_MOESM1_ESM.pdf]

Diversity of the bacterial community associated with Cliona lobata Hancock and Gelliodes pumila (Lendenfeld, 1887) sponges in the south-east coast of India

Ramu Meenatchi, Pownraj Brindangnanam, Hassan Saqib, Kumarasamy Rathna, Seghal G Kiran, Joseph Selvin

Project: reds-single

The SILVAngs Team
January 3, 2018

1 Introduction

The SILVAngs analysis pipeline is primarily targeting the analysis of large scale small- and large subunit (LSU/SSU) ribosomal RNA (rRNA) gene tag sequencing projects but can also be run on meta-genomes studies. Each project normally includes thousands to millions of reads from many different samples produced by massive parallel high-throughput "next generation" sequencing (NGS) technologies. Each read is aligned, quality checked, and classified based on the SILVA Reference alignment and taxonomy. Intuitive graphical outputs are provided for statistical information about the taxonomical distribution of the reads within and across samples. Interactive tax breakdowns are available for detailed inspection of the diversity in the samples.

Processing of the data is performed by five basic modules: *align, quality control, dereplication, clustering, and classification.*

In the first step the alignment is used to verify that each read is indeed, depending on the project, an LSU or SSU rRNA gene sequence. Ambiguous reads and reads that are not of the required rRNA gene type, will be rejected based on the alignment score and the alignment identity. This module also checks the sequence quality of each read and it filters out low-quality reads based on ambiguous bases, or too many homopolymers. The number of aligned bases, within the boundaries of the rRNA genes, is determined and sequences below a user defined minimal length cut-off are rejected.

After alignment and quality checks, the remaining sequences are dereplicated, clustered and classified. SILVAngs implements an approach similar to map and reduce. First all reads that are 100% identical (allowing overhangs) to another read are marked as replicate by the dereplication module. Next, the clustering module creates clusters of sequences with 98% sequence identity to each other (pairwise distance and single linkage clustering). The longest read in each cluster is selected as its reference. Finally, the classification module classifies all reference sequences. Currently, BLAST in combination with the SILVA SSU or LSU Ref datasets are used to classify the sequences. The resulting classification of the reference sequence of a cluster is mapped to all members of the respective cluster as well as their replicates. Sequences having an average BLAST alignment coverage and alignment identity of less than 93% will be considered as unclassified and assigned to the virtual taxonomical group "No Relative".

All results can be downloaded as CSV and SVG files. Aligned sequence data can be downloaded in the FASTA and ARB file formats.

If you consider this tool useful and use its results in a publication please consider citing Quast et al. (2013) for SILVA and the SILVAngs pipeline. The pipeline itself uses the following tools: SINA for the alignment of se-

quences (Pruesse et al., 2012), CD-HIT for the clustering of sequences (Li and Godzik, 2006), BLAST for the classification of sequences (Camacho et al., 2009), and KRONA for some parts of the visualisation of results (Ondov et al., 2011).

2 Material & Methods

Overview of method for analysis (this can roughly be paraphrased for the purpose of manuscripts and grants):

All sequence reads were processed by the NGS analysis pipeline of the SILVA rRNA gene database project (SILVAngs 1.3) (Quast et al., 2013). Each read was aligned using the SILVA Incremental Aligner (SINA SINA v1.2.10 for ARB SVN (revision 21008)) (Pruesse et al., 2012) against the SILVA SSU rRNA SEED and quality controlled (Quast et al., 2013). Reads shorter than 50 aligned nucleotides and reads with more than 2% of ambiguities, or 2% of homopolymers, respectively, were excluded from further processing. Putative contaminations and artefacts, reads with a low alignment quality (50 alignment identity, 40 alignment score reported by SINA), were idendified and excluded from downstream analysis.

After these inital steps of quality control, identical reads were identified (dereplication), the unique reads were clustered (OTUs), on a per sample basis, and the reference read of each OTU was classified. Dereplication and clustering was done using cd-hit-est (version 3.1.2; `http://www.bioinformatics.org/cd-hit`) (Li and Godzik, 2006) running in *accurate mode*, ignoring overhangs, and applying identity criteria of 1.00 and 0.98, respectively. The classification was performed by a local nucleotide BLAST search against the non-redundant version of the SILVA SSU Ref dataset (release 132; `http://www.arb-silva.de`) using blastn (version 2.2.30+; `http://blast.ncbi.nlm.nih.gov/Blast.cgi`) with standard settings (Camacho et al., 2009).

The classification of each OTU reference read was mapped onto all reads that were assigned to the respective OTU. This yields quantative information (number of individual reads per taxonomic path), within the limitations of PCR and sequencing technique biases, as well as, multiple rRNA operons. Reads without any BLAST hits or reads with weak BLAST hits, where the function "(% sequence identity + % alignment coverage)/2" did not exceed the value of 93, remain unclassified. These reads were assigned to the meta group "No Relative" in the SILVAngs fingerprint and Krona charts (Ondov et al., 2011).

This method was first used in the publications of Klindworth et al. (2013) and Ionescu et al. (2012)

References

Christiam Camacho, George Coulouris, Vahram Avagyan, Ning Ma, Jason Papadopoulos, Kevin Bealer, and Thomas Madden. BLAST+: architecture and applications. *BMC Bioinformatics*, 10(1):421, 2009. ISSN 1471-2105. doi: 10.1186/1471-2105-10-421. URL `http://www.biomedcentral.com/1471-2105/10/421`.

Danny Ionescu, Christian Siebert, Lubos Polerecky, Yaniv Y. Munwes, Christian Lott, Stefan Häusler, Mina Bižić-Ionescu, Christian Quast, Jörg Peplies, Frank Oliver Glöckner, Alban Ramette, Tino Rödiger, Thorsten Dittmar, Aharon Oren, Stefan Geyer, Hans-Joachim Stärk, Martin Sauter, Tobias Licha, Jonathan B. Laronne, and Dirk de Beer. Microbial and chemical characterization of underwater fresh water springs in the Dead Sea. *PLoS ONE*, 7(6):e38319, 06 2012. doi: 10.1371/journal.pone.0038319. URL `http://dx.doi.org/10.1371%2Fjournal.pone.0038319`.

Anna Klindworth, Elmar Pruesse, Timmy Schweer, Jörg Peplies, Christian Quast, Matthias Horn, and Frank Oliver Glöckner. Evaluation of general 16S ribosomal RNA gene PCR primers for classical and next-generation sequencing-based diversity studies. *Nucleic Acids Research*, 41(1):e1, 2013. doi: 10.1093/nar/gks808. URL `http://nar.oxfordjournals.org/content/41/1/e1.abstract`.

Weizhong Li and Adam Godzik. Cd-hit: a fast program for clustering and comparing large sets of protein or nucleotide sequences. *Bioinformatics*, 22(13):1658–1659, 2006. doi: 10.1093/bioinformatics/btl158. URL `http://bioinformatics.oxfordjournals.org/content/22/13/1658.abstract`.

Brian Ondov, Nicholas Bergman, and Adam Phillippy. Interactive metagenomic visualization in a web browser. *BMC Bioinformatics*, 12(1):385, 2011. ISSN 1471-2105. doi: 10.1186/1471-2105-12-385. URL `http://www.biomedcentral.com/1471-2105/12/385`.

Elmar Pruesse, Jörg Peplies, and Frank Oliver Glöckner. SINA: accurate high throughput multiple sequence alignment of ribosomal rna genes. *Bioinformatics*, 2012. doi: 10.1093/bioinformatics/bts252. URL `http://bioinformatics.oxfordjournals.org/content/28/14/1823`.

Christian Quast, Elmar Pruesse, Pelin Yilmaz, Jan Gerken, Timmy Schweer, Pablo Yarza, Jörg Peplies, and Frank Oliver Glöckner. The SILVA ribosomal RNA gene database project: improved data processing and web-based tools. *Nucleic Acids Research*, 41(D1):D590–D596, 2013. doi: 10.1093/nar/gks1219. URL `http://nar.oxfordjournals.org/content/41/D1/D590.abstract`.

Project Summary		
Project Name:	reds-single	
Porject Description:	Default configuration	
Sequence Type:	SSU	
Number of Samples:	1	
Number of Sequences:	695328	
Number of Rejected Sequences:	6978	(1.00 %)

Raw Sequence Information	
Min. Length:	301
Avg. Length:	301
Max. Length:	301

Aligned Sequence Information	
Min. Length:	4
Avg. Length:	301
Max. Length:	301

Quality Information (rejected by)	
Alignment BP Score:	11
Alignment Identity:	234
Alignment Score:	43
Ambiguous Bases:	-
Homopolymers:	6670
Quality:	-
Length:	20

Clustering Information		
Number of OTUs:	58048	(8.35 %)
Number of Clustered Sequences:	144168	(20.73 %)
Number of Replicates:	486134	(69.91 %)

Classification Information		
Number of Classified Sequences:	678517	(97.58 %)
Number of “No Relative”:	9833	(1.41 %)

Project Settings

Alignment	
SINA Version:	SINA v1.2.10 for ARB SVN (revision 21008)
Min Align. Identity (%):	50
Min Align. Score:	40
Min Basepair Score (%):	30

Quality Control	
Min. Sequence Quality (%):	30
Min. Length (aligned nuc.):	50
Max. Ambiguities (%):	2
Max. Homopolymers (%):	2

Clustering	
CD-Hit Version:	3.1.2
Min. OTU Identity (%):	98

Classification	
BLAST Version:	2.2.30+
Reference:	SILVA
Reference Version:	132
Similarity (%):	93

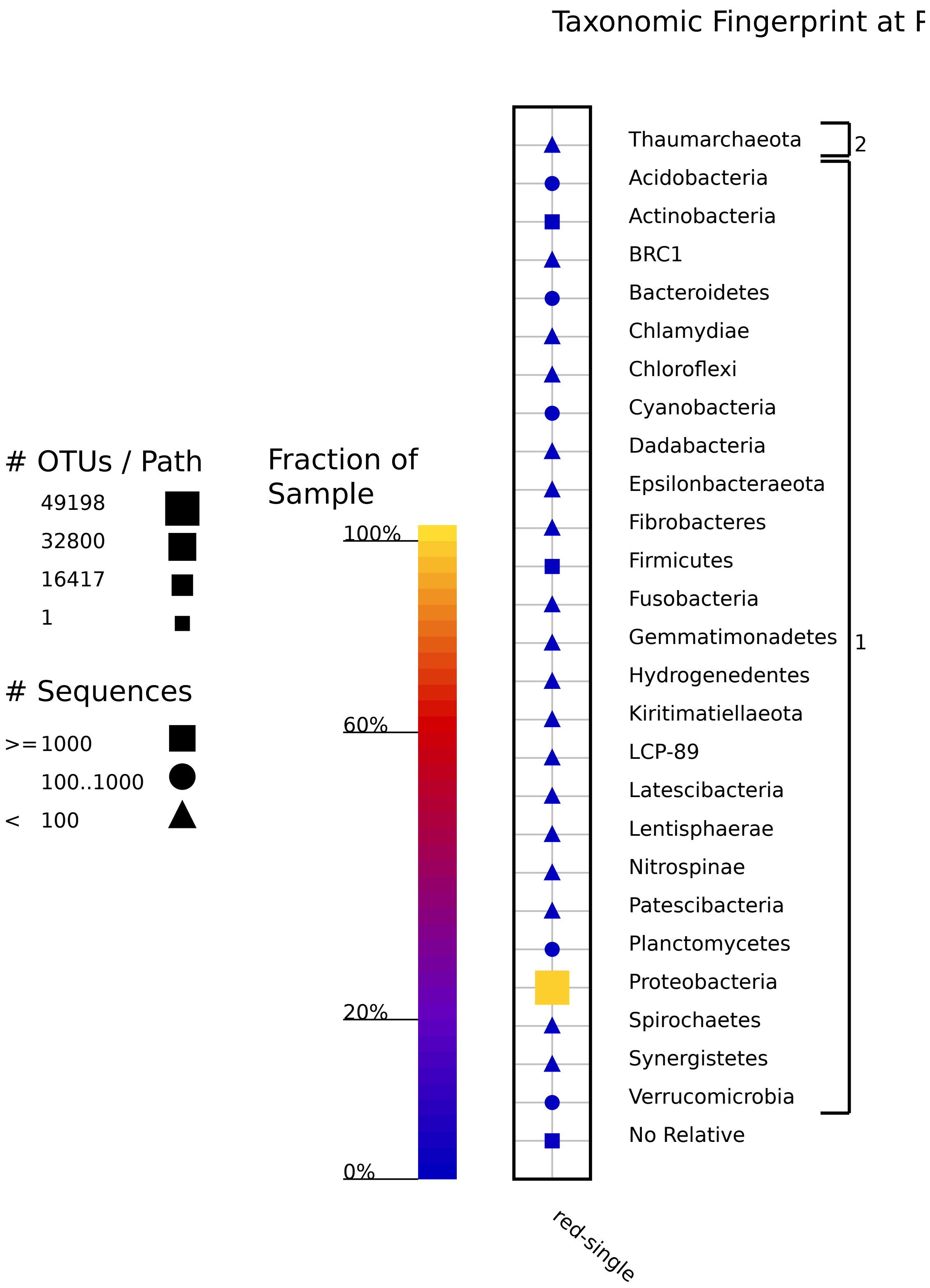
Taxonomic Fingerprint at F
OTUs / Path
49198
32800
16417
1
Sequences
>=1000
100..1000
< 100
Fraction of Sample
100%
60%
20%
0%
Thaumarchaeota
Acidobacteria
Actinobacteria
BRC1
Bacteroidetes
Chlamydiae
Chloroflexi
Cyanobacteria
Dadabacteria
Epsilonbacteraeota
Fibrobacteres
Firmicutes
Fusobacteria
Gemmatimonadetes
Hydrogenedentes
Kiritimatiellaeota
LCP-89
Latescibacteria
Lentisphaerae
Nitrospinae
Patescibacteria
Planctomycetes
Proteobacteria
Spirochaetes
Synergistetes
Verrucomicrobia
No Relative
2
1
red-single

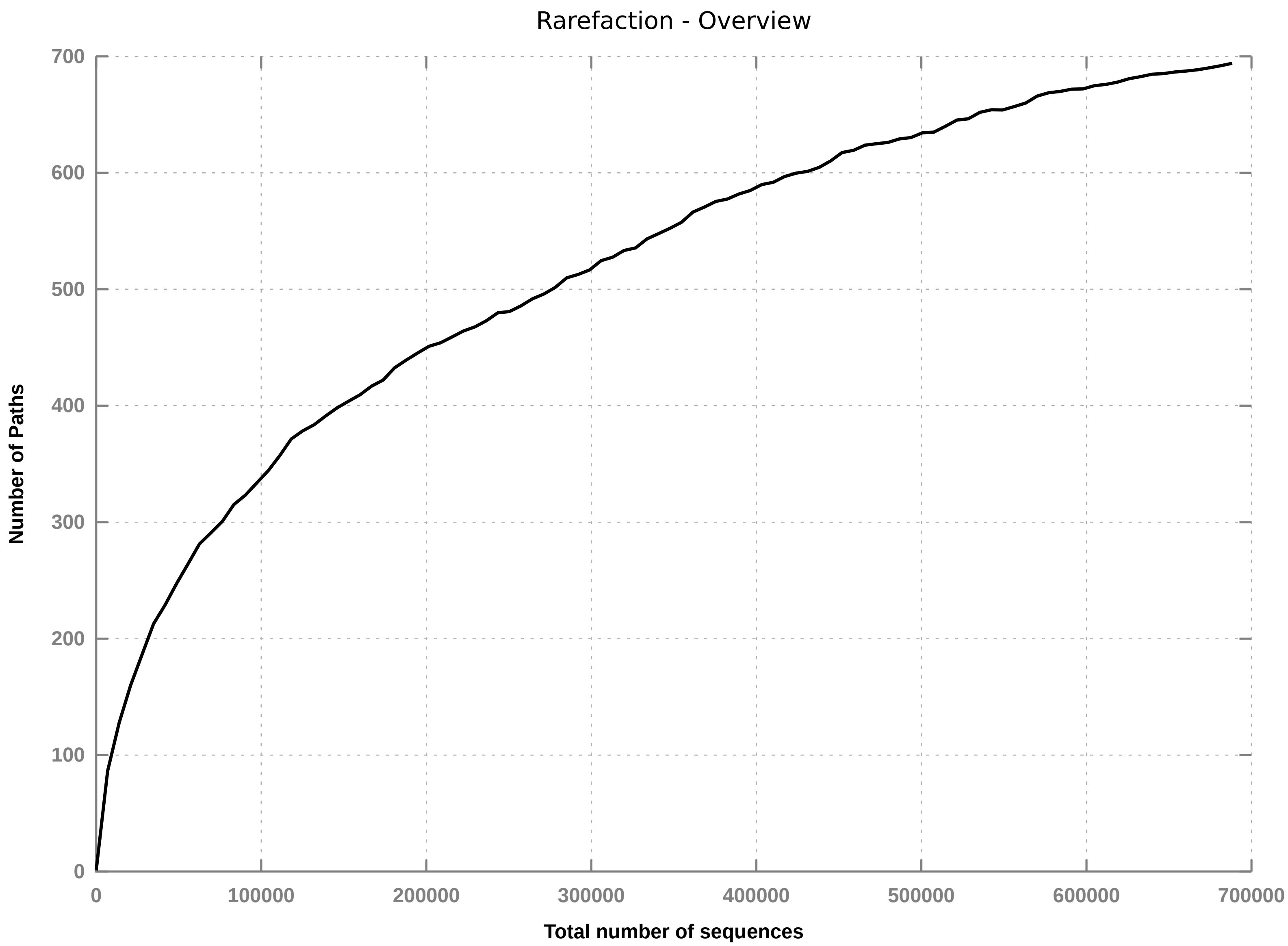
Rarefaction - Overview
Number of Paths
Total number of sequences
0
100
200
300
400
500
600
700
0
100000
200000
300000
400000
500000
600000
700000

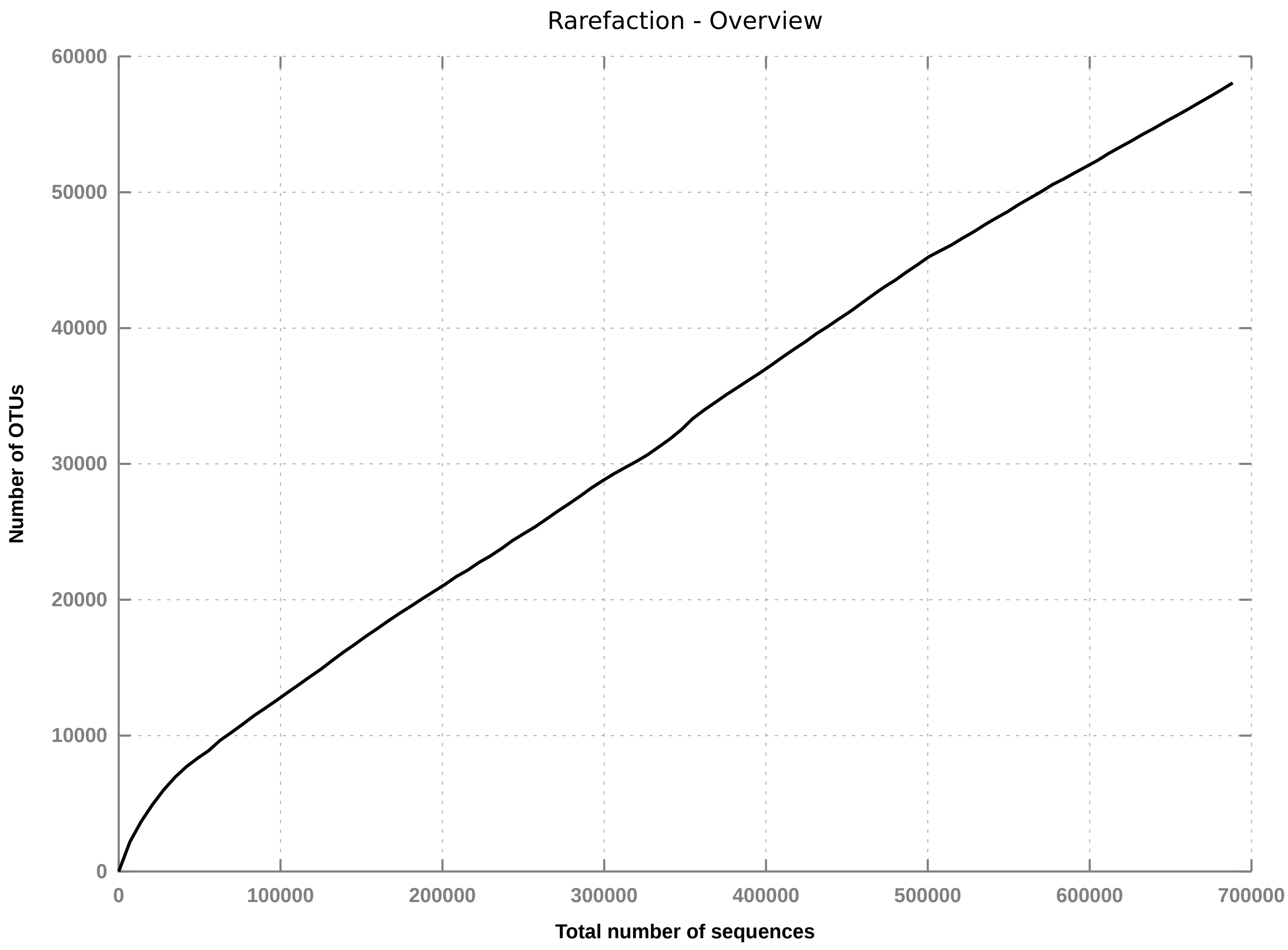
Rarefaction - Overview
Number of OTUs
Total number of sequences
0
10000
20000
30000
40000
50000
60000
0
100000
200000
300000
400000
500000
600000
700000

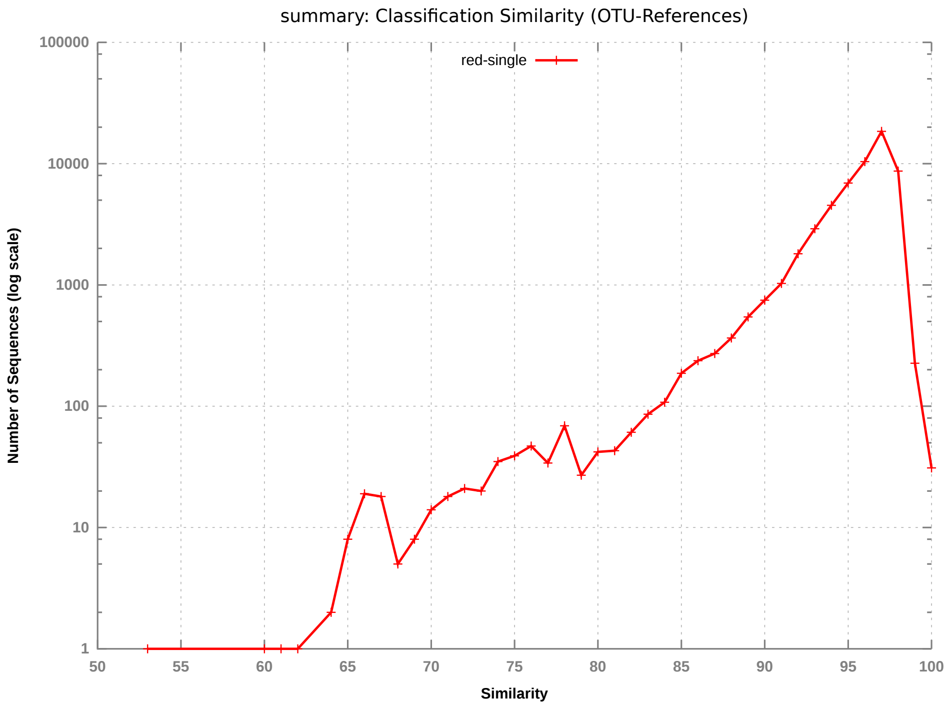
summary: Classification Similarity (OTU-References)
red-single
Number of Sequences (log scale)
Similarity
100000
10000
1000
100
10
1
50
55
60
65
70
75
80
85
90
95
100

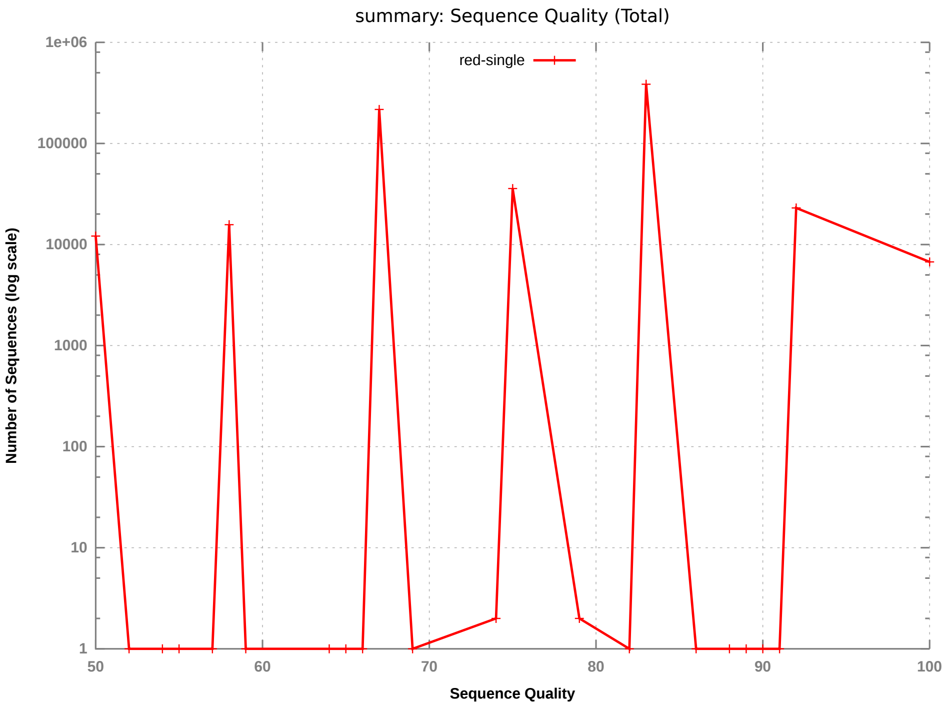
summary: Sequence Quality (Total)
red-single
Number of Sequences (log scale)
Sequence Quality
1e+06
100000
10000
1000
100
10
1
50
60
70
80
90
100

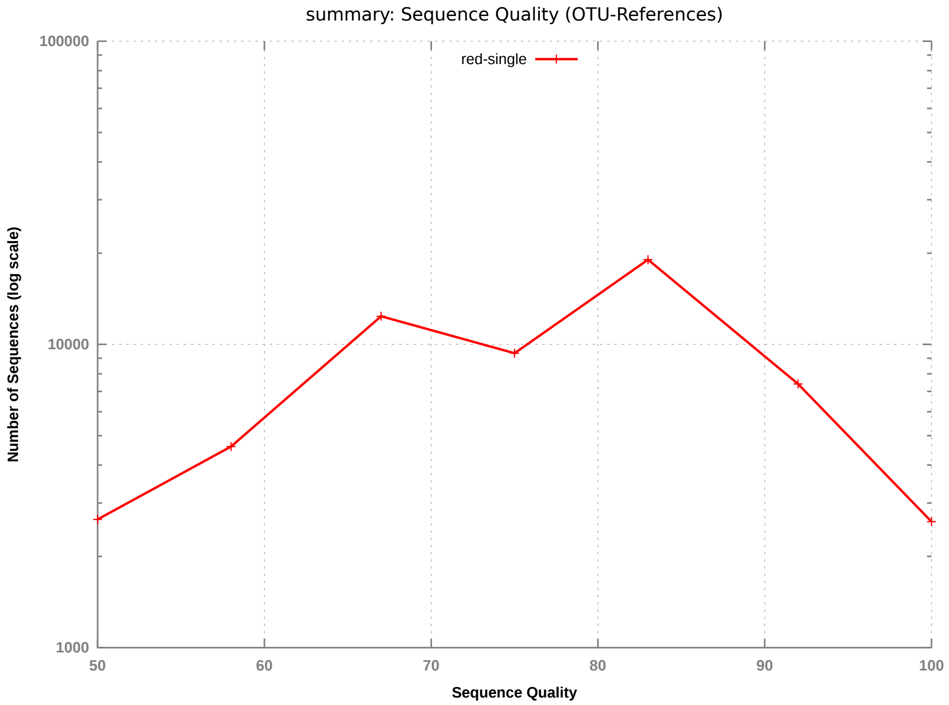
summary: Sequence Quality (OTU-References)
red-single
Number of Sequences (log scale)
Sequence Quality
100000
10000
1000
50
60
70
80
90
100

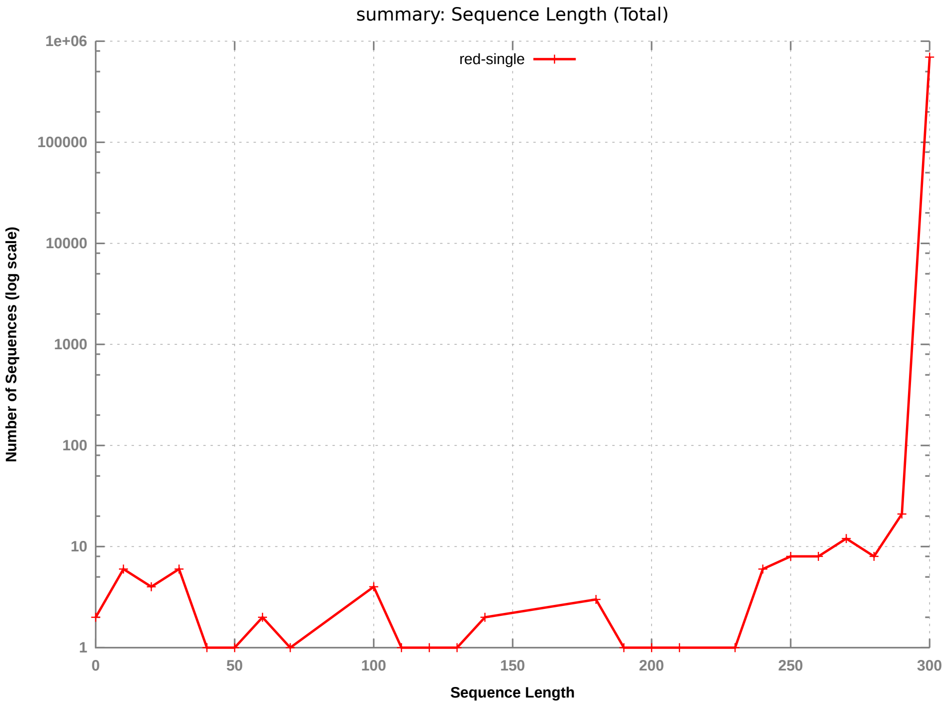
summary: Sequence Length (Total)
red-single
Number of Sequences (log scale)
Sequence Length
1e+06
100000
10000
1000
100
10
1
0
50
100
150
200
250
300

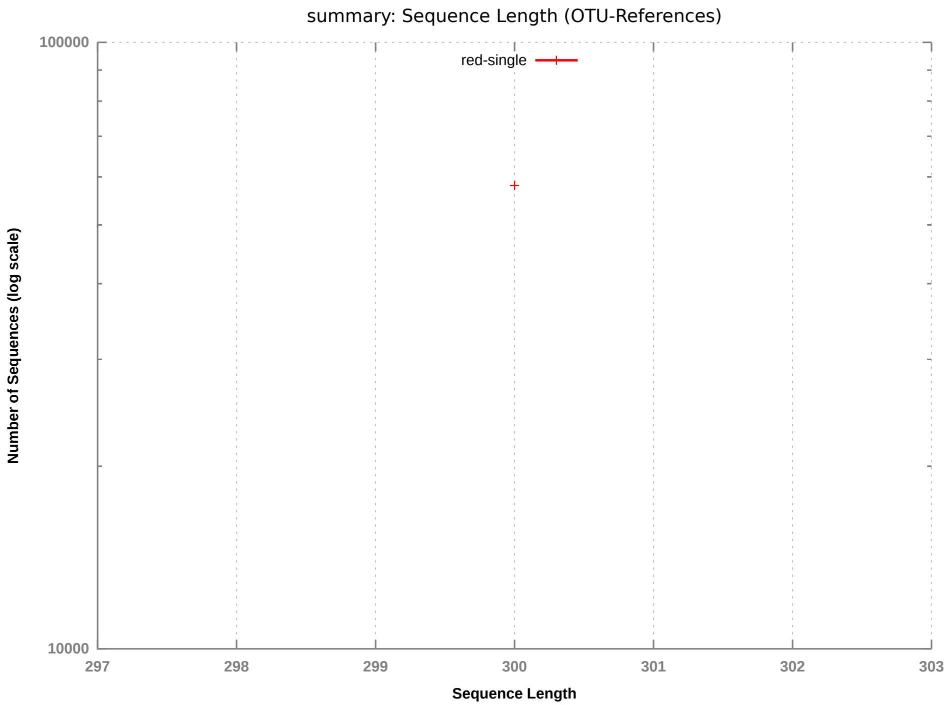
summary: Sequence Length (OTU-References)
red-single
Number of Sequences (log scale)
100000
10000
297
298
299
300
301
302
303
Sequence Length

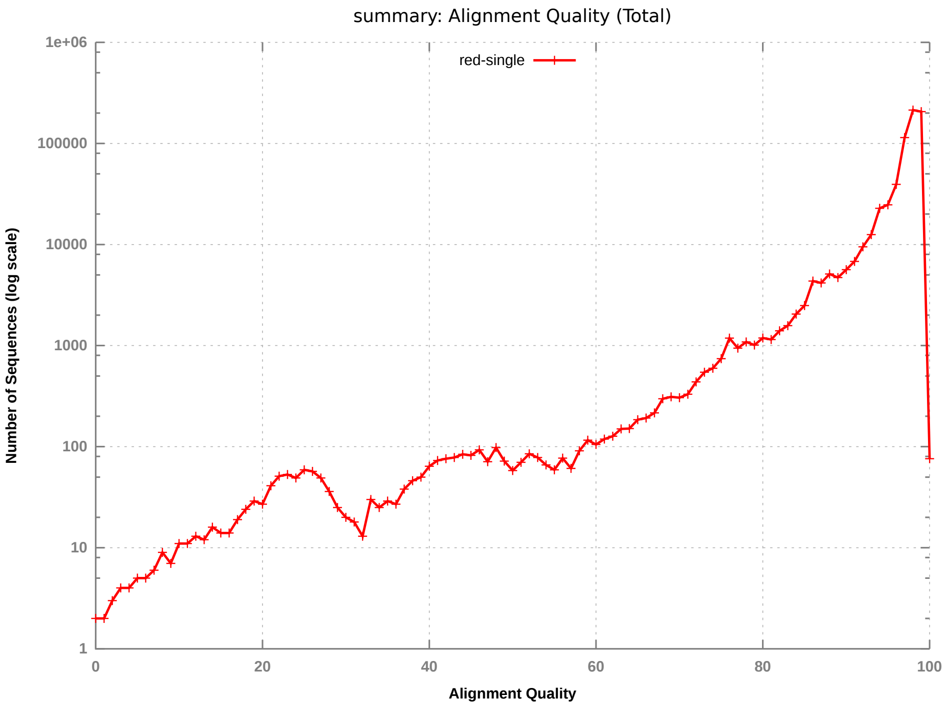
summary: Alignment Quality (Total)
red-single
Number of Sequences (log scale)
1e+06
100000
10000
1000
100
10
1
0
20
40
60
80
100
Alignment Quality

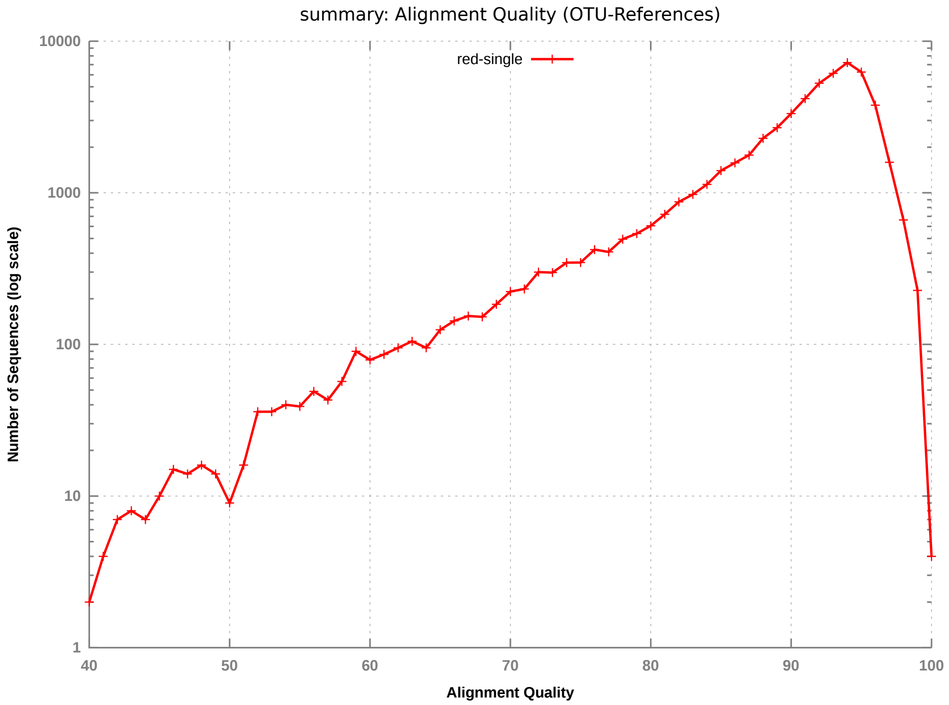
summary: Alignment Quality (OTU-References)
red-single
Number of Sequences (log scale)
10000
1000
100
10
1
40
50
60
70
80
90
100
Alignment Quality

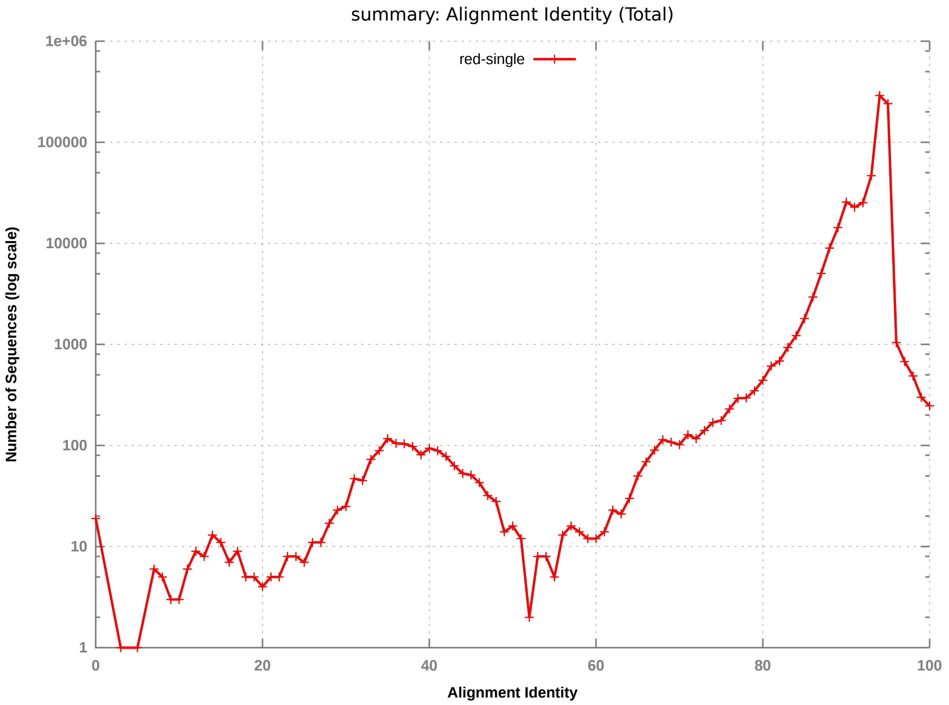
summary: Alignment Identity (Total)
red-single
Number of Sequences (log scale)
1e+06
100000
10000
1000
100
10
1
0
20
40
60
80
100
Alignment Identity

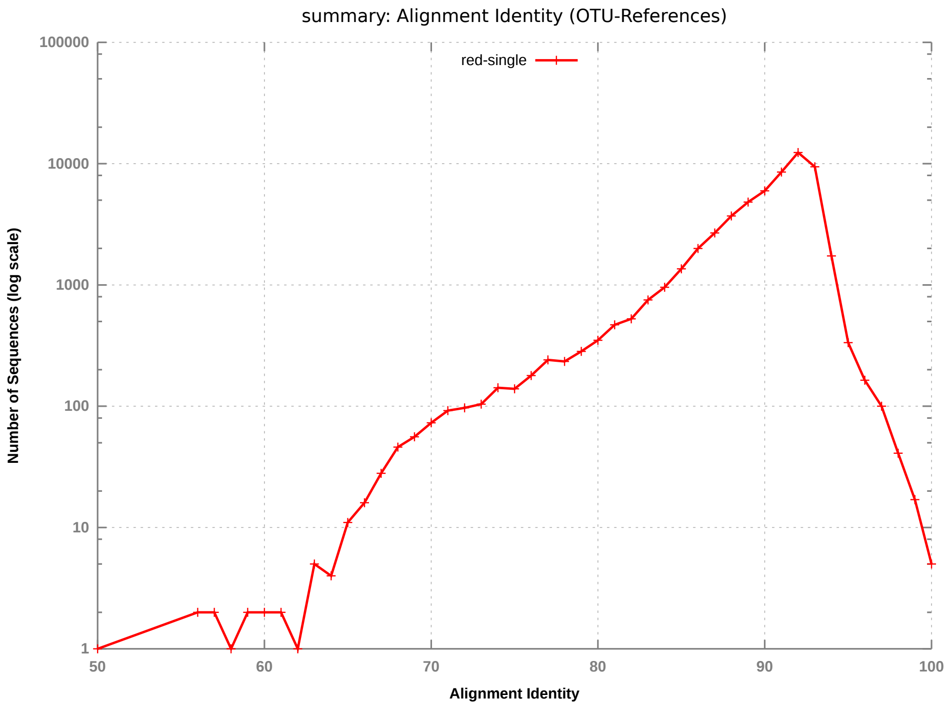

summary: Alignment Identity (OTU-References)
red-single
Number of Sequences (log scale)
100000
10000
1000
100
10
1
50
60
70
80
90
100
Alignment Identity
