# Supplementary material for: Diversity of a bacterial community associated with Cliona lobata Hancock and Gelliodes pumila (Lendenfeld, 1887) sponges on the South-East coast of India
Source: Sci Rep. 2020 Jul 14;10:11558. doi: 10.1038/s41598-020-67717-9 (PMC7360593; doi:10.1038/s41598-020-67717-9)

Diversity of the bacterial community associated with Cliona lobata Hancock and Gelliodes pumila (Lendenfeld, 1887) sponges in the south-east coast of India

Ramu Meenatchi, Pownraj Brindangnanam, Hassan Saqib, Kumarasamy Rathna, Seghal G Kiran, Joseph Selvin

Search:

all ↔

Total: 688350

- 6 + Max depth

- 11 + Font size

- + Chart size

Collapse

Snapshot

Link

?

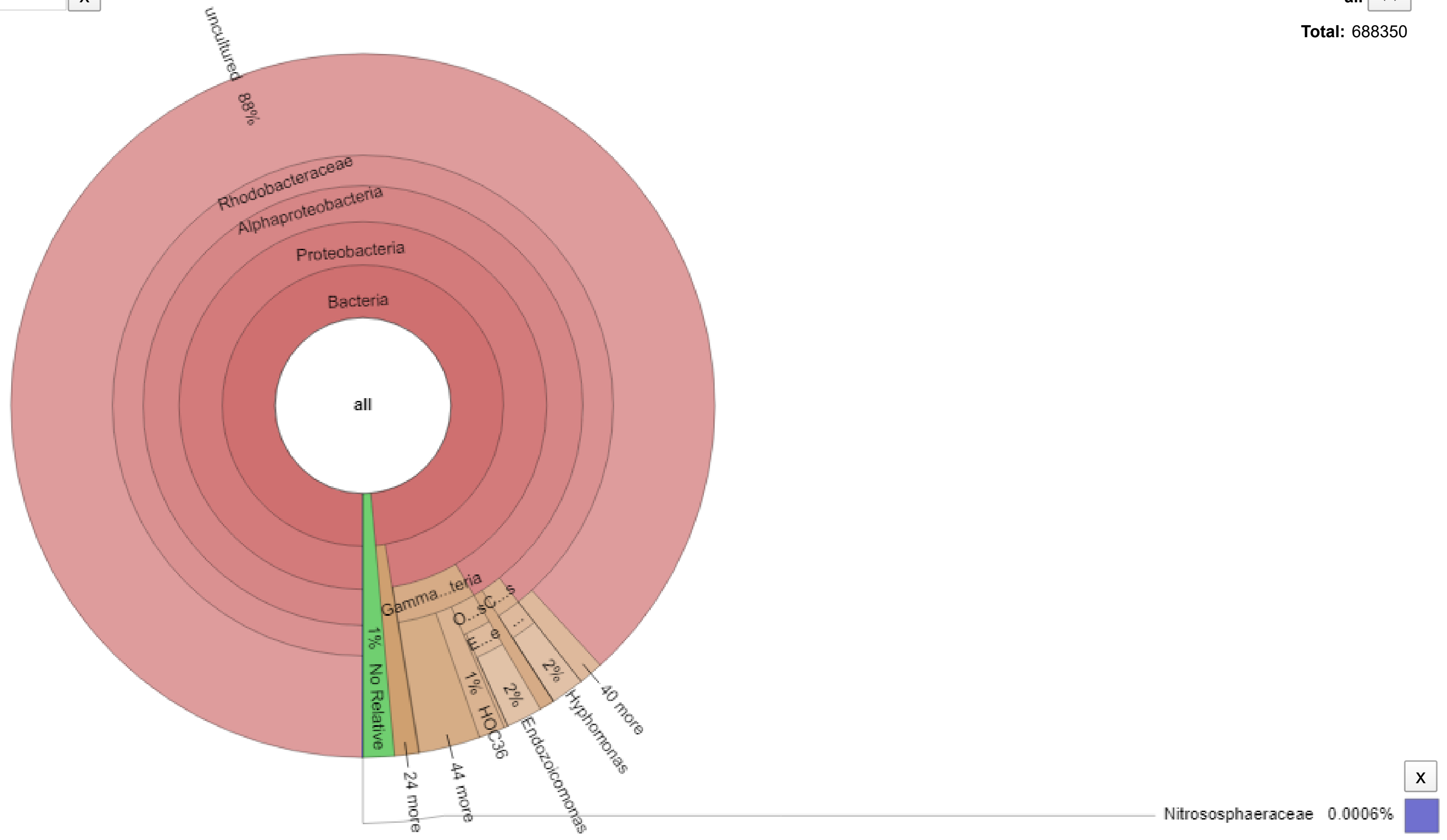

Supplement: Supplementary file 4 — Supplementary file4 (PDF 126 kb) [file 41598_2020_67717_MOESM4_ESM.pdf]
