# Supplementary material for: Diversity of a bacterial community associated with Cliona lobata Hancock and Gelliodes pumila (Lendenfeld, 1887) sponges on the South-East coast of India
Source: Sci Rep. 2020 Jul 14;10:11558. doi: 10.1038/s41598-020-67717-9 (PMC7360593; doi:10.1038/s41598-020-67717-9)
Supplement: Supplementary file 5 — Supplementary file5 (DOCX 317 kb) [file 41598_2020_67717_MOESM5_ESM.docx]

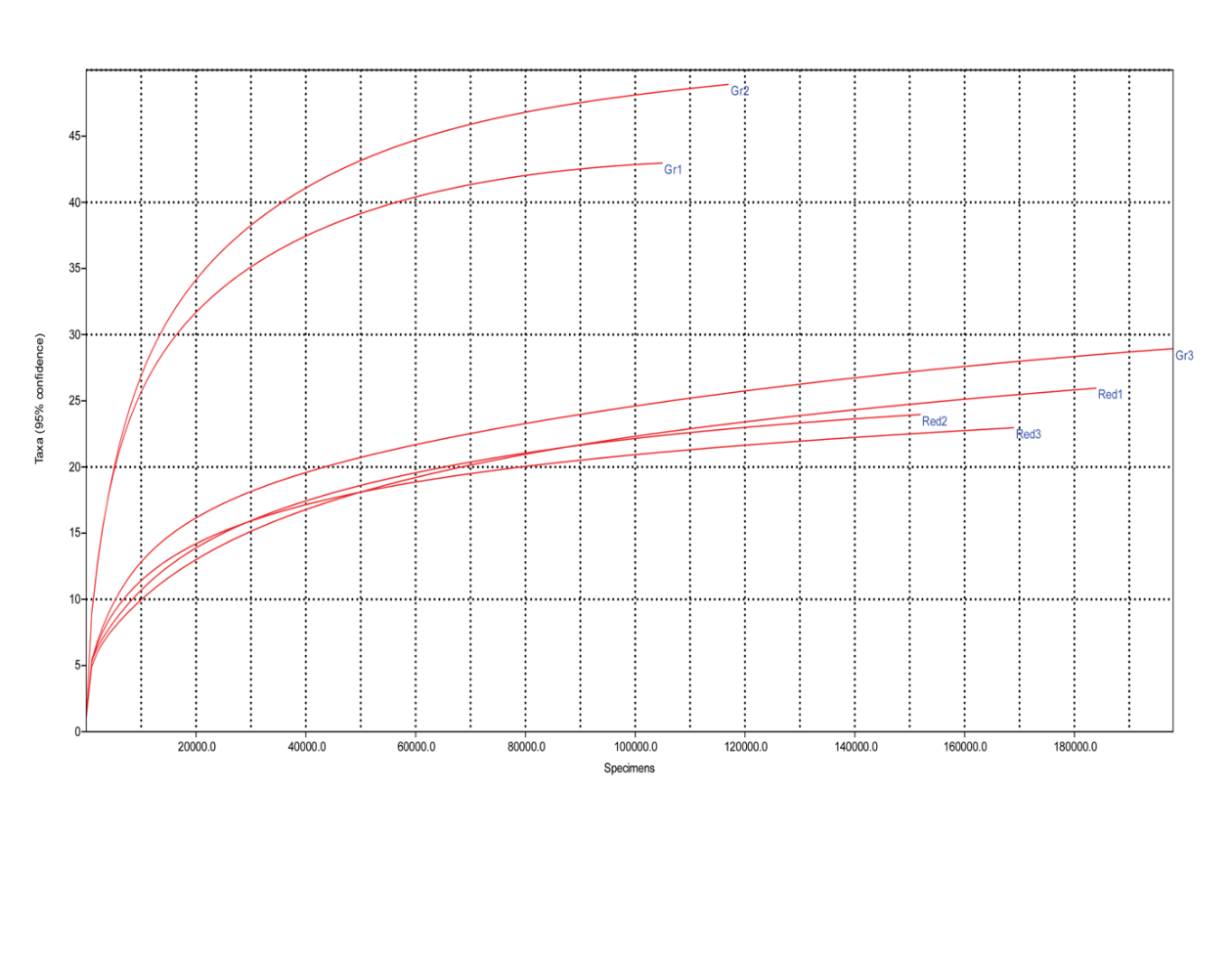


**Suppl. Fig. S1. Rarefaction curves for the sponge metagenome samples.** The curves for Red1, Red2, Red3 and Gr3 representing *Cliona lobata* and *Gelliodes pumila* show the number of operational taxonomic units (OTUs) as a function of number of sequences, indicating sampling completeness. Gr1 and Gr2 samples representing *G. pumila* shows plateau curve, indicating the partial bacterial profiling.

**Supplementary Table S1: BioSurfDB database search at species level for Class: *Alphaproteobacteria***

| **S.No** | **Species** | **BiosurfDB (strain)** | **Order** | **Family** |
| --- | --- | --- | --- | --- |
| 1. | *Labrenzia marina* | mano18 | *Rhodobacterales* | *Rhodobacteraceae* |
| 2. | *Pseudovibrio denitrificans* | unknown |  |  |
| 3. | *Roseibium hamelinense* | OCh 254 |  |  |
| 4. | *Methyloligella halotolerans* | C2 | *Rhizobiales* | *Rhodobiaceae* |
| 5. | *Methyloligella solikamskensis* | SK12 |  |  |
| 6. | *Skermanella aerolata* | KACC 11604 | *Rhodospirillales* | *Rhodobacteraceae* |

**Supplementary Table S2: KEGG orthology search at genus level**

| **S.No** | **Genus** | **KEGG Orthology** | **Antimicrobial resistance gene modules** | **Metabolism** |
| --- | --- | --- | --- | --- |
| 1. | *Altererythrobacter* | Xenobiotics biodegradation and metabolism | Beta-Lactam resistance modules, Vancomycin resistance modules, CAMP resistance modules, Multidrug resistance modules | Carbohydrates, amino acids, proteins, and nucleic acid metabolism |
| 2. | *Azospirillum* | Xenobiotics biodegradation and metabolism | Beta-Lactam resistance modules, Vancomycin resistance modules, CAMP resistance modules, Multidrug resistance modules | Carbohydrates, amino acids, proteins, and nucleic acid metabolism |
| 3. | *Ochrobactrum* | Xenobiotics biodegradation and metabolism | Beta-Lactam resistance modules, Vancomycin resistance modules, CAMP resistance modules, Multidrug resistance modules | Carbohydrates, amino acids, proteins, and nucleic acid metabolism |
| 4. | *Parvibaculum* | Xenobiotics biodegradation and metabolism | Beta-Lactam resistance modules, Vancomycin resistance modules, CAMP resistance modules, Multidrug resistance modules | Carbohydrates, amino acids, proteins, and nucleic acid metabolism |
| 5. | *Rhodovulum* | Xenobiotics biodegradation and metabolism | Beta-Lactam resistance modules, Vancomycin resistance modules, CAMP resistance modules, Multidrug resistance modules | Carbohydrates, amino acids, proteins, and nucleic acid metabolism |
| 6. | *Bartonella* | Xenobiotics biodegradation and metabolism | Beta-Lactam resistance modules, Vancomycin resistance modules, CAMP resistance modules, Multidrug resistance modules | Carbohydrates, amino acids, proteins, and nucleic acid metabolism |
| 7. | *Labrenzia* | Xenobiotics biodegradation and metabolism | Beta-Lactam resistance modules, Vancomycin resistance modules, CAMP resistance modules, Multidrug resistance modules | Carbohydrates, amino acids, proteins, and nucleic acid metabolism |
| 8. | *Pseudovibrio* | Xenobiotics biodegradation and metabolism | Beta-Lactam resistance modules, Vancomycin resistance modules, CAMP resistance modules, Multidrug resistance modules | Carbohydrates, amino acids, proteins, and nucleic acid metabolism |
| 9. | *Aminobacter* | Xenobiotics biodegradation and metabolism | Beta-Lactam resistance modules, Vancomycin resistance modules, CAMP resistance modules, Multidrug resistance modules | Carbohydrates, amino acids, proteins, and nucleic acid metabolism |
| 10. | *Gemmobacter* | Xenobiotics biodegradation and metabolism | Beta-Lactam resistance modules, Vancomycin resistance modules, CAMP resistance modules, Multidrug resistance modules | Carbohydrates, amino acids, proteins, and nucleic acid metabolism |
| 11. | *Hartmannibacter* | Xenobiotics biodegradation and metabolism | Beta-Lactam resistance modules, Vancomycin resistance modules, CAMP resistance modules, Multidrug resistance modules | Carbohydrates, amino acids, proteins, and nucleic acid metabolism |
| 12. | *Ochrobactrum* | Xenobiotics biodegradation and metabolism | Beta-Lactam resistance modules, Vancomycin resistance modules, CAMP resistance modules, Multidrug resistance modules | Carbohydrates, amino acids, proteins, and nucleic acid metabolism |
| 13. | *Phyllobacterium* | Xenobiotics biodegradation and metabolism | Beta-Lactam resistance modules, Vancomycin resistance modules, CAMP resistance modules, Multidrug resistance modules | Carbohydrates, amino acids, proteins, and nucleic acid metabolism |
| 14. | *Pleomorphomonas* | Xenobiotics biodegradation and metabolism | Beta-Lactam resistance modules, Vancomycin resistance modules, CAMP resistance modules, Multidrug resistance modules | Carbohydrates, amino acids, proteins, and nucleic acid metabolism |
| 15. | *Pseudovibrio* | Xenobiotics biodegradation and metabolism | Beta-Lactam resistance modules, Vancomycin resistance modules, CAMP resistance modules, Multidrug resistance modules | Carbohydrates, amino acids, proteins, and nucleic acid metabolism |
| 16. | *Rhodovulum* | Xenobiotics biodegradation and metabolism | Beta-Lactam resistance modules, Vancomycin resistance modules, CAMP resistance modules, Multidrug resistance modules | Carbohydrates, amino acids, proteins, and nucleic acid metabolism |
| 17. | *Thioclava* | Xenobiotics biodegradation and metabolism | Beta-Lactam resistance modules, Vancomycin resistance modules, CAMP resistance modules, Multidrug resistance modules | Carbohydrates, amino acids, proteins, and nucleic acid metabolism |
| 18. | *Chelativorans* | Xenobiotics biodegradation and metabolism | Beta-Lactam resistance modules, Vancomycin resistance modules, CAMP resistance modules, Multidrug resistance modules | Carbohydrates, amino acids, proteins, and nucleic acid metabolism |
| 19. | *Rhodovulum* | Xenobiotics biodegradation and metabolism | Beta-Lactam resistance modules, Vancomycin resistance modules, CAMP resistance modules, Multidrug resistance modules | Carbohydrates, amino acids, proteins, and nucleic acid metabolism |

* CAMP – cationic antimicrobial peptides
